# Isolation and characterisation of novel *Methanocorpusculum* species indicates the genus is ancestrally host-associated

**DOI:** 10.1186/s12915-023-01524-2

**Published:** 2023-03-22

**Authors:** James G. Volmer, Rochelle M. Soo, Paul N. Evans, Emily C. Hoedt, Ana L. Astorga Alsina, Benjamin J. Woodcroft, Gene W. Tyson, Philip Hugenholtz, Mark Morrison

**Affiliations:** 1 Faculty of Medicine, University of Queensland Frazer Institute, Translational Research Institute, Woolloongabba, 4102 Australia; 2 Centre for Microbiome Research, School of Biomedical Sciences, Queensland University of Technology (QUT), Translational Research Institute, Woolloongabba, 4102 Australia; 3 School of Chemistry and Molecular Biosciences and Australian Centre for Ecogenomics, University of Queensland, Saint Lucia, 4072 Australia; 4 Current Address: NHMRC Centre of Research Excellence (CRE) in Digestive Health, Hunter Medical Research Institute (HMRI), Newcastle, NSW Australia

**Keywords:** Methanogenic archaea, *Methanocorpusculum*, Comparative genomics, Metagenomics, Microbial cultivation, Marsupial

## Abstract

**Background:**

With an increasing interest in the manipulation of methane produced from livestock cultivation, the microbiome of Australian marsupials provides a unique ecological and evolutionary comparison with ‘low-methane’ emitters. Previously, marsupial species were shown to be enriched for novel lineages of *Methanocorpusculum*, as well as *Methanobrevibacter*, *Methanosphaera*, and *Methanomassiliicoccales*. Despite sporadic reports of *Methanocorpusculum* from stool samples of various animal species, there remains little information on the impacts of these methanogens on their hosts.

**Results:**

Here, we characterise novel host-associated species of *Methanocorpusculum*, to explore unique host-specific genetic factors and their associated metabolic potential. We performed comparative analyses on 176 *Methanocorpusculum* genomes comprising 130 metagenome-assembled genomes (MAGs) recovered from 20 public animal metagenome datasets and 35 other publicly available *Methanocorpusculum* MAGs and isolate genomes of host-associated and environmental origin. Nine MAGs were also produced from faecal metagenomes of the common wombat (*Vombatus ursinus*) and mahogany glider (*Petaurus gracilis*), along with the cultivation of one axenic isolate from each respective animal; *M. vombati* (sp. nov.) and *M. petauri* (sp. nov.).

**Conclusions:**

Through our analyses, we substantially expand the available genetic information for this genus by describing the phenotypic and genetic characteristics of 23 host-associated species of *Methanocorpusculum*. These lineages display differential enrichment of genes associated with methanogenesis, amino acid biosynthesis, transport system proteins, phosphonate metabolism, and carbohydrate-active enzymes. These results provide insights into the differential genetic and functional adaptations of these novel host-associated species of *Methanocorpusculum* and suggest that this genus is ancestrally host-associated.

**Supplementary Information:**

The online version contains supplementary material available at 10.1186/s12915-023-01524-2.

## Background

Methane is estimated to have ~ 82.5 times the global warming potential of carbon dioxide over a 20-year timescale [1, 2]. Animal agriculture is believed to be the largest source of anthropogenic methane emissions (95–109 Tg CH_4_/year), with ruminant livestock responsible for at least 80% (87–97 Tg CH_4_/year) of these emissions via feed digestion and associated microbial fermentation [3–5]. Typically, herbivorous diets consisting of foliage or lignin-rich plant products favour microbial communities with increased methanogen diversity and abundance, largely due to the increased availability of substrates through the bacterial hydrolysis and fermentation of plant polysaccharides [6]. Interestingly, some of Australia’s native marsupial herbivores appear to be ‘low-methane’ emitters. For instance, kangaroos and wallabies (members of the Macropodidae family) are foregut fermenters and eruct less methane (when corrected for digestible energy intake) compared to ruminant livestock when reared on the same diet [7]. Further work has similarly shown two kangaroo species, *Macropus fuliginosus* and *M. rufus*, to produce lower concentrations of methane compared to ruminant animals [8].

Several surveys of the foregut digesta contents from these animals suggested methanogen communities are much smaller in both relative and absolute abundances compared to ruminants and in some cases undetectable [9–11]. Some methanogen genera are common to the ruminant and macropodid foregut including *Methanobrevibacter*, *Methanosphaera*, and *Methanomassiliicoccus* (originally classified as *Thermoplasmatales*) [11, 12]. However, there is evidence that members of these genera are distinct between ruminants and marsupials. For example, marsupial *Methanosphaera* species have smaller genomes and broader methanogenic substrate profiles than their ruminant counterparts [12, 13]. Novel lineages of methanogens have also been observed in metagenomics analysis of the gut microbiome of koala (*Phascolarctos cinereus*) and southern hairy-nosed wombat (*Lasiorhinus latifrons*), which showed uncharacterised *Methanocorpusculum* species in both animals, with a greater relative abundance (2.14%) in the wombat compared to koala (0.11%) [14], suggesting that this lineage may represent a prominent group of methanogens in marsupials. A recent analysis of host-associated archaeal diversity using 16S rRNA gene amplicon data further supports that *Methanocorpusculum* is a significant archaeal genus that resides within the digestive tract of diverse animal species [15]. However, there is currently a paucity of knowledge about the ecology and evolution of host-associated *Methanocorpusculum* lineages due to a lack of available genomes and cultured isolates.

Here, we describe the expansion of the phylogenetic and functional understanding of host-associated *Methanocorpusculum* species using axenic isolates recovered from the common wombat (*Vombatus ursinus*) and mahogany glider (*Petaurus gracilis*), as well as MAGs produced from native Australian herbivores and other publicly available datasets. Using these MAGs and isolate genomes, we subsequently perform the first comparative genomic analysis of the genus *Methanocorpusculum* and identify host-specific genetic adaptations of novel *Methanocorpusculum* species.

## Results

### *Methanocorpusculum* detected in Australian marsupials

**Fig. 1:**
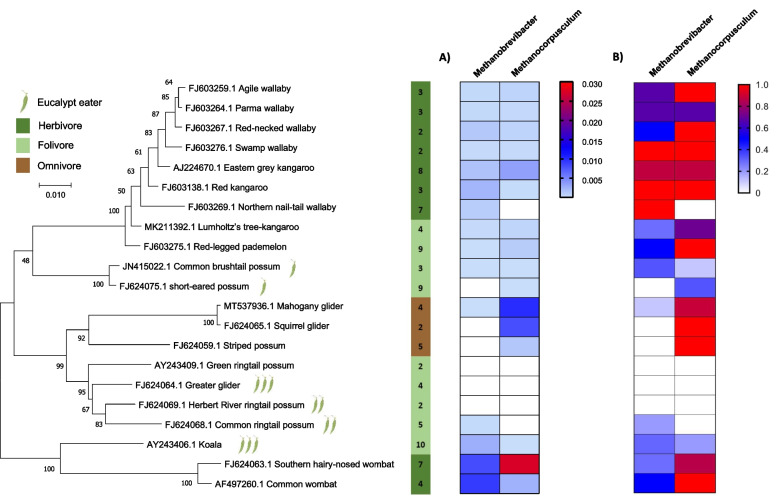
Methanogen profiles detected in marsupial species with 16S rRNA amplicon sequencing. The phylogenetic tree was built using the exon 28 of the von Willebrand factor (vWF) of the respective species available from the NCBI nucleotide database. MEGA-X [19] with MUSCLE was used to align the genes, and phylogeny was inferred using maximum likelihood and 1000 bootstrap replications. **A** The average relative abundance of respective OTUs and empty cells indicates no methanogen signal was detected. **B** The prevalence of respective OTUs for each species. The host diet composition is displayed as per legend, adapted from Shiffman et al. [14]. The number of samples is displayed to the right of the respective species

To further explore the prevalence of *Methanocorpusculum* in the faecal microbiome of Australian marsupials, the faecal DNA of 23 marsupial species (*n* = 102) was screened using universal 16S rRNA amplicon sequencing (Additional file 1: Table S1). Only two archaeal OTUs were detected: one *Methanobrevibacter* OTU closely related to *Methanobrevibacter gottschalkii* HO [16] (98.81% sequence identity) and one OTU with 99.2% sequence identity to *Methanocorpusculum labreanum* Z [17, 18]. Of the marsupial faecal samples, 68% (69/102) contained at least one detectable methanogen OTU (Additional file 1: Table S1). Only 25% (26/102) of samples contained both OTUs, though the majority of these (58%) belonged to the Macropodidae. Interestingly, the average relative abundance of *Methanocorpusculum* was significantly lower in the marsupial samples that also contained *Methanobrevibacter*, 0.10 ± 0.22% compared to 0.99 ± 1.56% (*p* = 0.0013; Fig. 1A, Additional file 1: Table S1), suggesting there may be competition between these two groups of methanogens.

This competition is especially evident in the wombats with *Methanocorpusculum* detected in all wombat samples except for one southern hairy-nosed wombat, which had a high abundance of *Methanobrevibacter* (5.52%; Fig. 1B). Furthermore, the wombat samples with *Methanobrevibacter* contained a lower average relative abundance of *Methanocorpusculum* (0.04 ± 0.06%; *n* = 4) compared to those without (2.85 ± 2.20%; *n* = 7; Fig. 1B). The southern hairy-nosed wombat also contained the greatest abundance of *Methanocorpusculum* (2.72 ± 2.35%; Fig. 1A), while the common wombat had a substantially lower abundance (0.25 ± 0.24%). Similarly, all squirrel gliders (4/4) and 89% (8/9) of mahogany gliders contained *Methanocorpusculum*, and only a single mahogany glider contained *Methanobrevibacter* (Fig. 1B). The mahogany glider samples contained the second greatest average abundance of *Methanocorpusculum* at 1.0 ± 1.2%, with the greatest individual faecal abundance of 3.26% (Fig. 1A, Additional file 1: Table S1). The Macropodidae contained a lower abundance of *Methanocorpusculum* at 0.11 ± 0.22% potentially due to a higher prevalence of *Methanobrevibacter* (70%; Fig. 1A, B)*.* The faecal samples from koalas contained both methanogen OTUs, though only three samples were positive for *Methanobrevibacter* and two for *Methanocorpusculum*. The average abundance of *Methanobrevibacter* was greater than that of the kangaroo and wallaby; however, this was attributed to a single koala sample with 2.6%, greater than any other marsupial sample except for the wombat methanogen communities.

### Isolation of novel host-associated *Methanocorpusculum* species enriched in marsupials

Given the high abundance and prevalence of *Methanocorpusculum* in the common wombat (CW) and mahogany glider (MG), as well as their high methane production in faecal enrichments relative to other marsupials (Additional file 2: Fig. S1), efforts were made to obtain pure cultures of *Methanocorpusculum* from the enrichments. Cultures supplemented with a combination of CO_2_/H_2_ and sodium acetate produced high microbial growth coupled with methane production. Amplicons produced from the CW and MG enrichment cultures both clustered within the order Methanomicrobiales, forming a deep lineage to other available *Methanocorpusculum* 16S rRNA sequences (Additional file 2: Fig. S2). Both enrichment cultures were representatives of the *Methanocorpusculum* OTU (100% sequence identity) identified in the marsupial faecal samples (see above; Fig. 1) and distinct from other cultured *Methanocorpusculum* spp., with 97% identity to the 16S rRNA of *Methanocorpusculum labreanum*. Single colonies were picked after ~ 4 weeks of incubation at 37 °C. Axenic cultures are henceforth referred to as *Methanocorpusculum* sp. MG for the mahogany glider isolate and *Methanocorpusculum* sp. CW153 for the common wombat isolate.

The whole-genome sequences for *Methanocorpusculum* sp. CW153 and MG are near complete (97.69 and 98.01%, respectively) with low contamination (1.96 and 1.31%, respectively; Table 1). Compared to the three other previously published *Methanocorpusculum* isolate genomes, all of which are from environmental sources, the genomes of CW153 and MG are larger and also possess a greater number of predicted coding genes according to IMG JGI based annotations (Table 1; Additional file 1: Table S2). Taxonomic classification using GTDB-Tk showed CW153 to be the same species as *Methanocorpusculum* sp001940805 represented by Phil4, a MAG produced from a faecal sample of a southern hairy-nosed wombat [14]. Strain MG was only classified to the genus level and thus likely represents a novel *Methanocorpusculum* species. Indeed, the pairwise average nucleotide identity (ANI) of CW153 and MG was only 88%, indicating that the two isolates represent distinct species of host-associated *Methanocorpusculum*, according to the operational ≥ 95% ANI threshold commonly used for species demarcation [20] (Additional file 1: Table S3).

**Table 1:** Summary of genome statistics for cultured *Methanocorpusculum* strains. Strain designation, estimated completeness, estimated contamination, genome size, number of contigs, N50, guanine-cytosine (GC) content, coding density, number of tRNAs out of the 20 canonical amino acids, 5S rRNA count, 16S rRNA count, 23S rRNA count, geographical location, isolation source, and GTDB classification are shown for each available cultured isolate. Only cultured isolates with available genomic information were included

	***M. labreanum***	***M. parvum***	***M. bavaricum***	***M.*** sp. CW153	***M.*** sp. MG
**Strain**	Z	XII	SZSXXZ	CW153	MG
**Completeness (%)**	99.54	98.21	98.21	97.69	98.01
**Contamination (%)**	0	0	0.66	1.96	1.31
**Coverage**	34	213	–	177.1	199.461
**Genome size (Mb)**	1.8	1.71	1.7	1.94	2.03
**Contigs (no.)**	1	47	34	47	59
**N50 (bp)**	1,804,962	74,097	161,459	164,607	87,295
**GC content (%)**	50	51.4	51.4	53.3	52
**Genes (no.)**	1816	1732	1748	1962	2087
**Coding density (%)**	88.4	90.2	88.3	88.4	89.3
**tRNAs (/20)**	18	18	18	18	18
**5S rRNA**	3	1	3	1	1
**16S rRNA**	3	1	1	1	1
**23S rRNA**	3	1	1	1	1
**Geography**	USA	Germany	Germany	Australia	Australia
**Isolation source**	Tar pit lake sediment	Anaerobic sour whey digester	Wastewater pond mud sediment	*Vombatus ursinus*	*Petaurus gracilis*
**GTDB classification**	*Methanocorpusculum labreanum*	*Methanocorpusculum parvum*	*Methanocorpusculum parvum*	*Methanocorpusculum* sp001940805	*Methanocorpusculum*;s
**Isolation reference**	Zhao et al. [17]	Zellner et al. [21]	Zellner et al. [22]	This study	This study

MG and CW153 stained Gram-negative and presented as pleomorphic cells, ~ 0.5 to 1.5 μm in diameter (Additional file 2: Fig. S3B). Viable cells of the two strains were auto-fluorescent at 420 nm (470 nm emission), due to the presence of the reduced form of cofactor *F*_420_ (Additional file 2: Fig. S3B) [23, 24]. Transmission electron micrographs (TEM) of MG and CW153 again showed pleomorphism, with both possessing a thin cell wall and singular membrane with no obvious capsule-like structures (Additional file 1: Fig. S3A).

### Recovery of *Methanocorpusculum*-associated MAGs from diverse animal hosts

To further characterise novel host-associated lineages of *Methanocorpusculum*, 130 MAGs assigned to the genus *Methanocorpusculum* were successfully recovered from publicly available metagenomes (Additional file 1: Table S4), with 24 of these MAGs being high-quality (HQ; ≥ 90% completeness, ≤ 5% contamination). These MAGs were combined with nine MAGs produced from southern hairy-nose wombats and mahogany gliders from this study, four human-associated MAGs [25], four environmental MAGs [26], one MAG produced from a wombat [14], 15 MAGs produced from ruminants [27], one MAG from a chicken [28], and 10 other MAGs identified as *Methanocorpusculum* on the NCBI genome database (Additional file 1: Table S5).

**Fig. 2:**
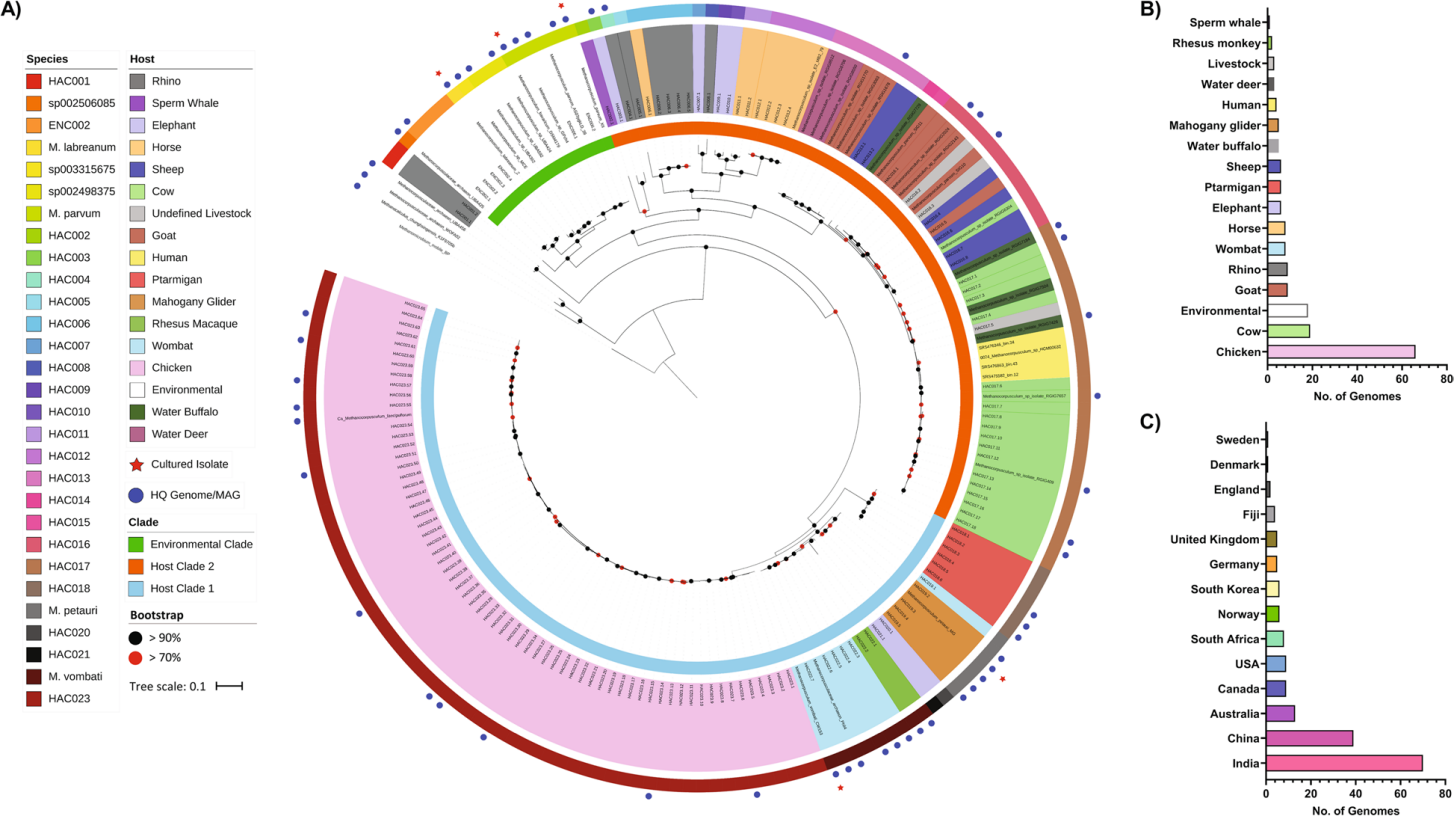
Phylogenetic distribution of *Methanocorpusculum* MAGs and isolate genomes. **A** Concatenated archaeal marker gene files were produced using GTDB-Tk (v.1.3.0) [29], with *Methanomicrobium mobile* BP used as the outgroup. Phylogeny was inferred using FastTree (v2.1.10) [30] and visualisation by iToL (https://itol.embl.de/). MAGs and isolate genomes of ≥ 50% completeness and ≤ 10% contamination were included, and HQ MAGs were identified by blue circles. Cultured isolates were identified by a red star. Bootstrap values are shown by the red (≥ 0.7) and black (≥ 0.9) circles. All MAGs and isolate genomes from environmental sources were identified as ‘environmental’ under the host description. **B** The host distribution and **C** geographical distribution of *Methanocorpusculum* genomes

A genome tree comprising the resultant 176 *Methanocorpusculum* genomes was constructed using a concatenated set of 122 archaeal marker genes that shows a striking diversity of host-associated *Methanocorpusculum* species (Fig. 2). There are at least six environment-associated (ENC) and 23 host-associated (HAC) *Methanocorpusculum* species (≥ 95% AAI), of which 17 are novel (Fig. 2, Additional file 1: Table S5) as of GTDB release 06-RS202. MAGs recovered from rhinoceros, elephant, and horse samples contained the greatest diversity, comprising nine species (Fig. 2; HAC001, 002–012, 020–021). Two MAGs from rhinoceros produced an outlying clade compared to all other *Methanocorpusculum* genomes (Fig. 2; HAC001) likely representing a novel Methanocorpusculaceae genus. One MAG recovered from a sperm whale was phylogenetically distinct but grouped closest with HAC003 and HAC004 recovered from elephant and rhinoceros, respectively. The MAGs recovered from domesticated (i.e. sheep, cows, and goats) and wild (i.e. water buffalo and water deer) ruminants were assigned to five species (HAC013–HAC017). Additionally, four human-derived MAGs were also assigned to HAC017 (Fig. 2). The MAGs recovered from ptarmigan represent a divergent *Methanocorpusculum* species (Fig. 2; HAC018), potentially resulting from the geographic isolation of this avian species. *Methanocorpusculum* MAGs and genomes were recovered from two Australian marsupials and separated into two distinct species: those produced from mahogany gliders (*M. petauri* sp. nov.; see below) and those from wombats (*M. vombati* sp. nov.; see below), except for one wombat MAG which clustered with *M. petauri*. Two MAGs produced from rhesus macaque were also assigned to *M. vombati*, and two MAGs recovered from elephants represent two species (HAC020, HAC021) closely related to but distinct from *M. vombati*. HAC023 is the most highly represented species with 66 MAGs, accounting for the most represented host (Fig. 2B; chicken) and geographical location (Fig. 2C; India), though this is likely a sampling artefact due to a large number of chicken metagenomes analysed compared to other animals.

### *Methanocorpusculum* genotypes cluster according to the host environment

**Fig. 3:**
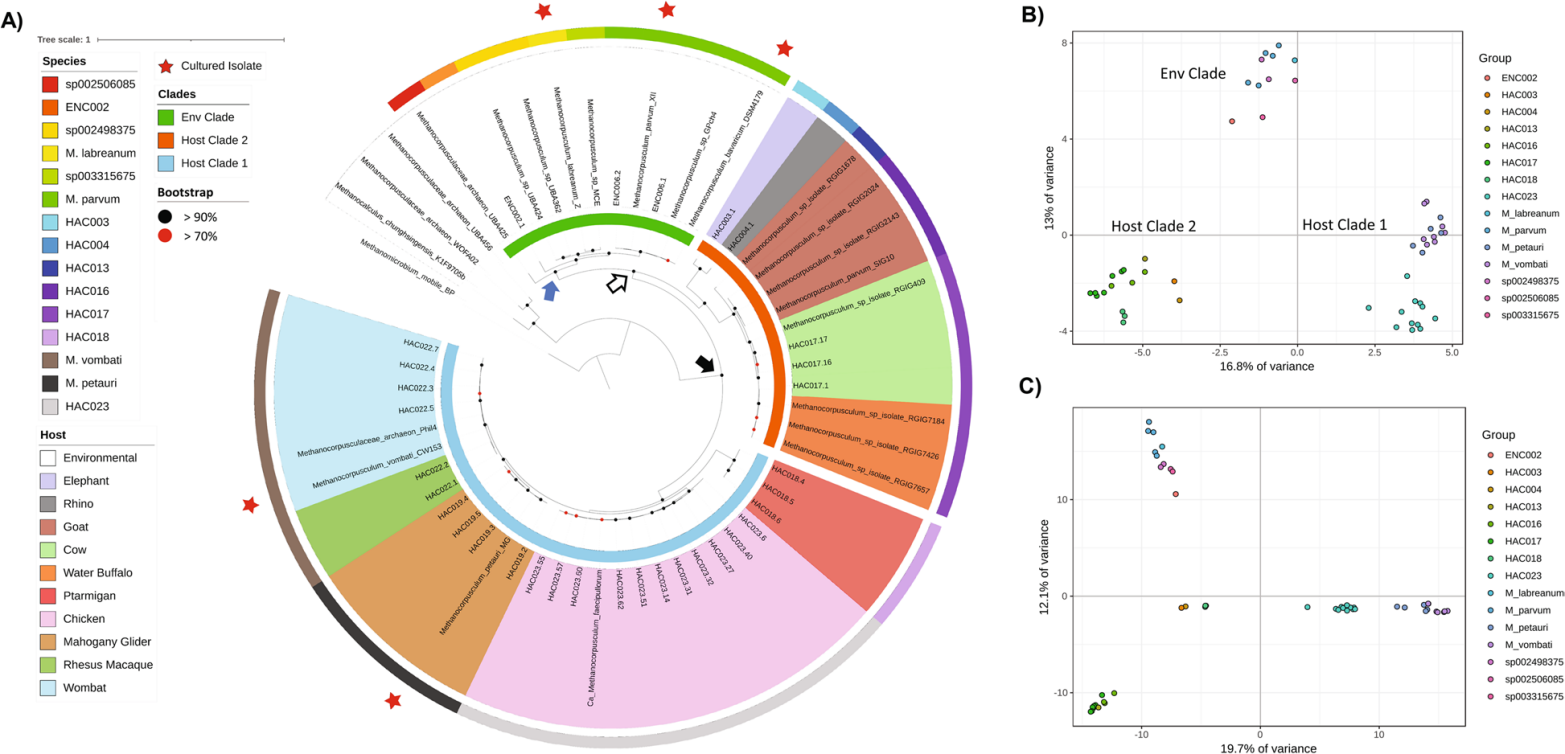
Phylogenetic and genotypic distribution of high-quality *Methanocorpusculum* MAGs and isolate genomes. **A** Phylogenetic tree of concatenated archaeal marker gene files was produced using GTDB-Tk (v.1.3.0) [29], with *Methanomicrobium mobile* BP used as the outgroup. Phylogeny was inferred using FastTree (v2.1.10) [30] and visualisation by iToL (https://itol.embl.de/). MAGs and isolate genomes of ≥ 90% completeness and ≤ 5% contamination were included. Cultured isolates are identified by a red star. Bootstrap values are shown by the red (≥ 0.7) and black (≥ 0.9) circles. The black arrow denotes the common ancestor of *Methanocorpusculum*, the white arrow denotes the common ancestor of the Env clade and host clade 2, and the blue arrow denotes the common ancestor of the Env clade. **B**, **C** The genetic variance based on the presence/absence of KO and ortholog annotation, respectively. Gene annotations and PCA plots were generated using EnrichM (v0.4.15, https://github.com/geronimp/enrichM) with the ‘--ko’ (**B**) and ‘--orthologs’ (**C**) analyses. Genomes are coloured according to species, as per the legend

To explore the genetic potential of *Methanocorpusculum*, 52 high-quality MAGs and isolate genomes representing 14 *Methanocorpusculum* species were compared (Fig. 3A, Additional file 1: Table S5). These included five ENC and nine HAC species, noting that no species had both environmental and host-associated representatives. AAI confirmed the separation of these species based on an operational definition (≥ 95% AAI; Additional file 2: Fig. S4). Analysis of the core and pan genomes, as defined by Chaudhari et al. [31], showed 8290 genes contributing to the *Methanocorpusculum* pangenome and only 149 core genes (~ 1.8%; Additional file 2: Fig. S5A). When the 11 ENC and 41 HAC genomes were analysed separately, the number of core genes for the ENC genomes increased to 890 out of 3097 (~ 29% of the pangenome). However, the core genes for the HAC genomes only increased to 179 out of 6559 (~ 2.7% of the pangenome), again showing the diversity of the HAC species (Additional file 2: Fig. S5B-C). It is worth noting the HAC maintained a significantly smaller core genome when 10 genomes were randomly sampled from both groups; 533 for the HAC compared to 918 for the ENC (*P* < 0.0001, Additional file 2: Fig. S5B-C). As expected, genomes from individual species contained a greater number of core genes (848–1441), although the MAGs from ptarmigan contained a smaller number of core genes, likely attributed to the comparatively smaller genome size and number of coding genes (Additional file 2: Fig. S6).

Based on a well-supported phylogeny, we infer that the ancestor of the genus *Methanocorpusculum* was host-associated and that this trait was lost in one line of descent (arrowed in Fig. 3A). This environmental clade currently comprises six species, including *M. labreanum* and *M. parvum*, and effectively splits host-associated species into two distinct clades—host clade 1 (four species found in birds, marsupials, and one species of old-world monkey) and host clade 2 (five species found in pachyderms and ruminants; Fig. 3A). These two distinct clades likely correspond to the dominant host-associated *Methanocorpusculum* clades recently identified by Thomas et al. [15] using 16S rRNA sequencing. This phylogenetic separation is reflected in predicted functional differences between the clades including pathways involved in amino acid, carbohydrate, energy, and lipid metabolism, as well as membrane transport, signalling and cellular processes, and genetic information processing (Fig. 3B, C; Additional file 1: Table S6). Genes encoding homocitrate synthase, homoisocitrate, and homoaconitase were significantly enriched in the environmental *Methanocorpusculum* spp. Interestingly, the ptarmigan genomes were deemed phylogenetically distinct (Fig. 3A) but share functional features with genomes from the ruminant group (Fig. 3B), suggesting these two separate lineages show some convergence in their adaptation to a similar habitat provided by their host. Indeed, further evidence of host-specific adaptations are reflected in the gene annotations lacking a current functional assignment (Fig. 3C).

### Host-associated *Methanocorpusculum* species have unique metabolic potential

According to KO annotations, 413 genes were significantly differentially enriched between the *Methanocorpusculum* species (Additional file 1: Table S7). Most of these KOs were metabolic (53%), ~ 25% transport-associated, and ~ 12% genetic information processing proteins. KOs assigned to the metabolism of cofactors and vitamins, amino acid metabolism, cell motility and defence, and transport proteins were significantly differentially enriched between the host groups (Additional file 1: Table S8, Additional file 2: Figs. S7-S12).

**Fig. 4:**
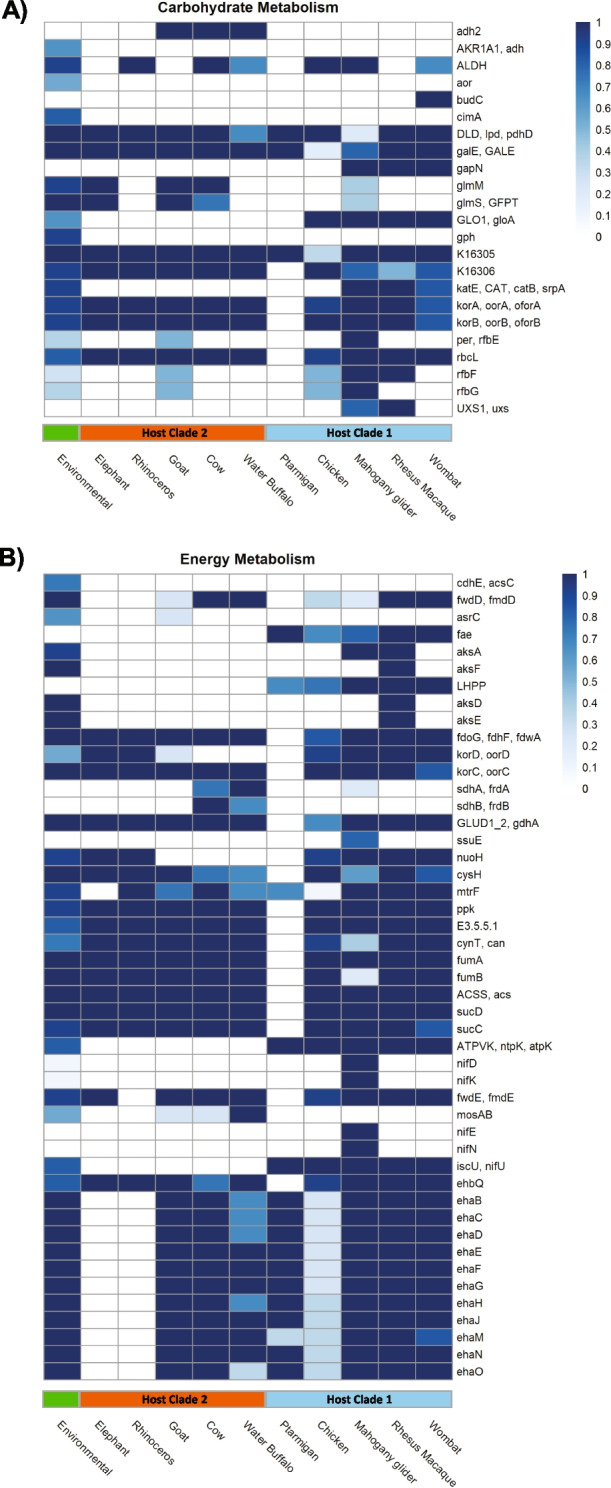
Differential enrichment of carbohydrate and energy metabolism-associated genes in *Methanocorpusculum*. KO annotation and statistical analyses were performed using the ‘annotate’ and ‘enrichment’ functions of EnrichM (v0.4.15; https://github.com/geronimp/enrichM). Genomes were grouped by host species and compared by Fisher’s exact test, where KOs with corrected *p* values of < 0.05 were retained and considered significant. Heatmap values are colour-coded according to the legend and represent the proportion of respective genomes for a given host group. The *Methanocorpusculum* are also labelled as environmental clade (green), host clade 2 (orange), and host clade 1 (blue), as per Fig. 3. **A** Carbohydrate metabolism. **B** Energy metabolism

In terms of genes encoding functions for methanogenesis and energy production, the environmental genomes were significantly enriched for alcohol dehydrogenase (*adh*) AKR1A1 (K00002; Fig. 4A). This gene was described in *M. parvum* and inferred to be involved in the use of short-chain alcohols as alternative reductants in CO_2_-dependent methanogenesis (Fig. 4A) [32]. The absence of this gene in host-associated *Methanocorpusculum* species suggests that they are unable to use short-chain alcohols through this pathway. However, the *M. vombati* (HAC022) genomes were significantly enriched for a predicted meso-butanediol dehydrogenase (*budC*) and may allow for the utilisation of meso-2,3-butanediol, (S)-acetoin and/or (S,S)-butane-2,3-diol for the NADH-dependent production of hydrogen (Fig. 4A) [33]. Additionally, the genomes from ruminant hosts (HAC016-017) were enriched for a different alcohol dehydrogenase family (*adh2*), which has been characterised by the use of 2-propanol by *Gordonia* [34].

Some species of *Methanocorpusculum* are also enriched for genes which could support additional or alternative pathways for growth. For example, HAC003, *M. vombati*, and *M. petauri* encode genes annotated as benzoyl-CoA reductase subunit A (*badF*; Additional file 2: Fig. S12), which catalyses intermediate steps in benzoate degradation and is closely related to 2-hydroxyglutaryl-CoA dehydratase of amino acid fermenting *Gottschalkia*. Although *badF* is a key marker gene for aromatic compound metabolism for many bacteria [35, 36], our *Methanocorpusculum*-affiliated MAGs and isolate genomes do not possess genes with confirmed or putative capacity for the complete metabolic pathway. As such, while our results raise the intriguing possibility of there being “ancillary” metabolic scheme(s) in these species, coupled or uncoupled from methanogenesis and/or growth, more detailed studies are needed to functionality validate these predictions. The *M. vombati* genomes were significantly enriched for *adh1*, with the protein sequence sharing 56% similarity (*E* = 6e−157) to the predicted phosphonoacetaldehyde reductase of the bacterium *Natronincola peptidivorans* (Additional file 2: Fig. S12). Phosphoenolpyruvate phosphomutase (PPM) and phosphonopyruvate decarboxylase (PPD) were also enriched in the marsupial genomes (Additional file 2: Fig. S12), with all three genes involved in phosphonate metabolism. These genes are found within a single gene cluster in the *Methanocorpusculum vombati* CW153 genome flanked by genes with similarity to bacterial transposases (CDS358) and, as such, may have been acquired through horizontal gene transfer.

Furthermore, multiple genes of bacterial origin were found to be enriched in the marsupial lineages. For instance, *M. petauri* was significantly enriched for KOs associated with nitrogen assimilation (*nifDEKNU*, *nifHD1/2*; Fig. 4B, Additional file 2: Fig. S6), which also look to be of bacterial origin. Additionally, the marsupial *Methanocorpusculum* species were differentially enriched for specific CAZymes, including glycosyltransferase (GT) families 2, 39, and 83 (Additional file 2: Fig. S13). *M. vombati* specifically encodes for a greater number of GT4 and GT66, as well as carbohydrate-binding module (CBM) 44 that has been shown to bind both cellulose and xyloglucan (Additional file 2: Fig. S13) [37]. Comparatively, *M. petauri* encodes for a greater number of GT8, with the genome of *M. petauri* MG specifically encoding for the greatest number of GT2, as well as GT11, GT111, and GT10 (Additional file 2: Fig. S13). These unique CAZymes show the greatest similarity to bacterial protein sequences, suggesting they may also be of bacterial origin.

### Marsupial-associated *Methanocorpusculum* isolates have simplified substrate utilisation

**Fig. 5:**
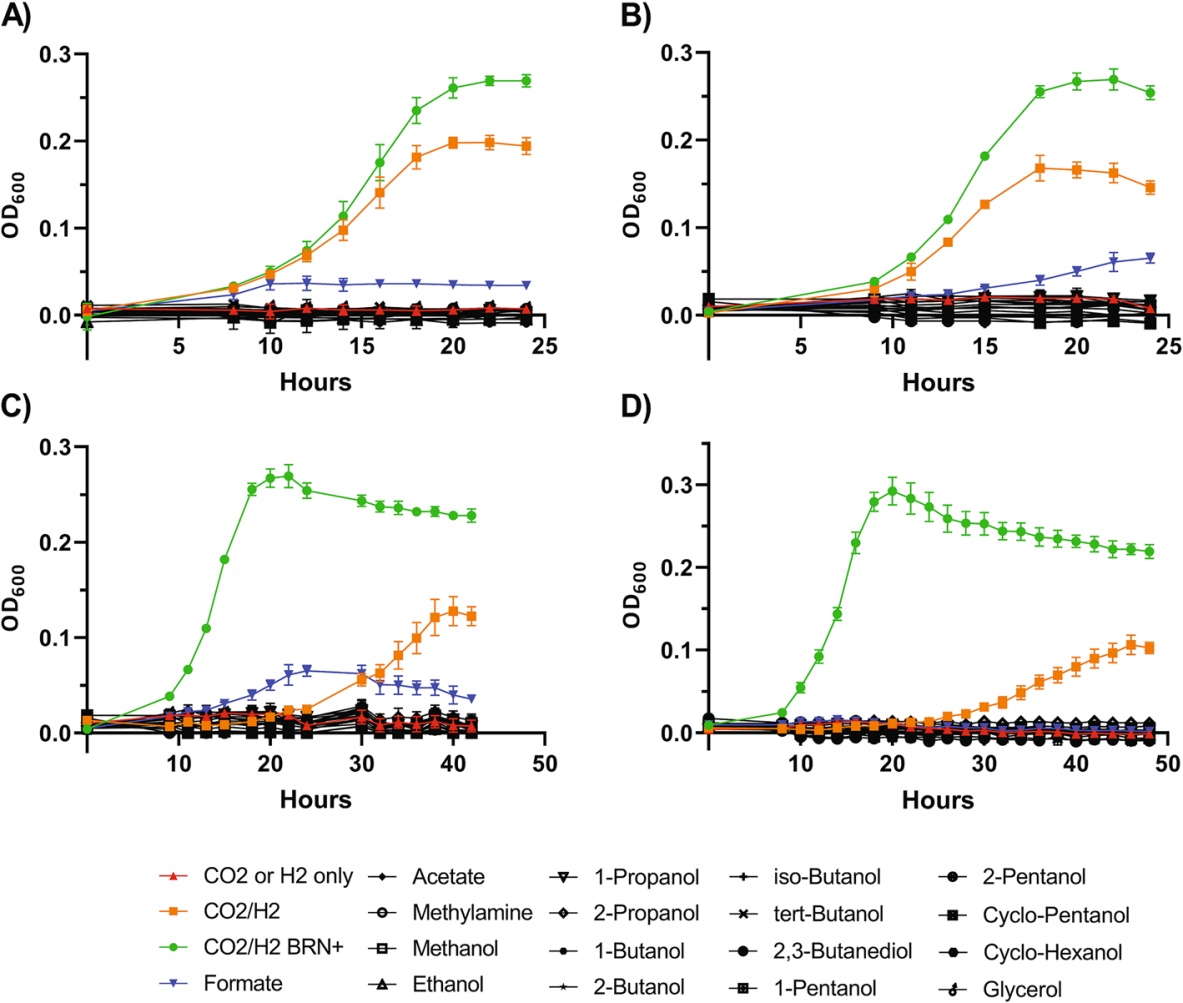
Primary in vitro substrate utilisation profile of *M. vombati* CW153 and *M. petauri* MG. Strains were grown using BRN-RF10 medium without added sodium formate and sodium acetate at 37 °C with 1% (v/v) supplementation of each substrate, as listed in the legend. CO_2_/H_2_ was used for the positive control. CO_2_/H_2_ with ‘BRN+’ (i.e. with sodium formate and sodium acetate added) was used to show growth in basal BRN-RF10 medium. **A**, **B** *M. vombati* CW153 cultured with a headspace of CO_2_ and H_2_, respectively. **C**, **D** *M. petauri* MG cultured with a headspace of CO_2_ and H_2_, respectively. CO_2_ and H_2_ alone were used for negative controls. Growth was measured by optical density at 600 nm (OD_600_) at ~ 2 h interval

As expected, and like their environmental counterparts, both *Methanocorpusculum vombati* and *petauri* could use CO_2_ and H_2_ for growth [17, 38], but the removal of sodium acetate and sodium formate from the basal media significantly reduced the maximum yield of both strains (Fig. 5). While formate is a likely substrate for methanogenesis via formate dehydrogenase mediated activation, acetate is unlikely to be used for methanogenesis, instead as a source for central carbon assimilation, as shown for *M. parvum* [32, 38].

Despite the lack of *M. parvum adh* homologue in our cultured isolates, another type of *adh* was predicted to be present. As such, it was unclear whether these new host-associated *Methanocorpusculum* spp. could utilise short-chain alcohols for growth via an alternative, currently uncharacterised pathway(s). To that end, both *M. vombati* CW153 and *M. petauri* MG were cultured using a basal medium prepared to contain various short-chain alcohols to determine their potential to perform alcohol-dependent methanogenesis. Contrary to the environmental *Methanocorpusculum* isolates, neither *M. vombati* nor *M. petauri* showed significant growth with short-chain alcohols as substrates for methanogenesis, even with prolonged incubation (500 h; data not shown). It is worth noting that *M. petauri* grown with a headspace of CO_2_ and 1-butanol supplementation did produce a statistically significant increase in yield (OD_600_ = 0.022) after ~ 200 h of incubation; however, such a small increase in yield suggests CO_2_ and 1-butanol are likely not viable substrates for *M. petauri* (Additional file 2: Fig. S14). Such findings suggest that both *M. vombati* and *M. petauri* are effectively incapable of growth with short-chain alcohols as alternative substrates for methanogenesis, consistent with the absence of an *adh* homologue in these species (AKR1A1; Fig. 4).

## Discussion

The genus *Methanocorpusculum* has long been recognised as “environmental” methanogens, principally isolated from soils and hydrocarbon-rich bogs [17, 21]. However, there are sporadic reports of 16S rRNA gene amplicons affiliated with *Methanocorpusculum* from stool samples of various animal species [22, 39]. Recently, Thomas et al. [15] showed the presence of *Methanocorpusculum*-associated sequences across diverse lineages of animal hosts, suggesting that this genus represents an important but largely uncharacterised host-associated group of methanogens. We have expanded on previous studies [9–11, 14] to show the presence of *Methanocorpusculum* in the faecal microbiomes of a wide variety marsupial species, including a relatively high abundance in wombat and glider species. Furthermore, we have isolated two host-associated representatives of this family from a common wombat and mahogany glider. Based on our phylogenetic, AAI, and comparative genomic analyses, these isolates represent two novel host-associated species of *Methanocorpusculum*, for which we propose the names *M. vombati* (strain CW153) and *M. petauri* (strain MG), respectively. These strains constitute the second and third host-associated isolates for this genus, after *M. aggregans* BU5 isolated from a buffalo [40], and the first two for which genomic information is available.

Shiffman and colleagues [14] successfully recovered a MAG from the faecal metagenome of a southern hairy-nosed wombat, *Methanocorpusculum* sp. Phil4, representing the first host-associated genome of *Methanocorpusculum*. Subsequently, *Methanocorpusculum* MAGs have been produced as part of several metagenomic studies of both human and non-human hosts [25, 27, 28, 41]. Through our analysis, we have further expanded the number of available *Methanocorpusculum* MAGs to include more than 20 distinct species from the faecal microbiomes of various mammalian and avian animals. Interestingly, the phylogenetic placement and genomic profile of each species were consistent except for HAC018, which grouped phylogenetically with host clade 1 but genetically with host clade 2 (Fig. 3). Like the hoatzin, ptarmigan have evolved a prominent crop for the microbial fermentation of a highly herbivorous diet that may have induced similar genetic adaptations to those observed in the *Methanocorpusculum* recovered from ruminant hosts [42, 43].

Environmental *Methanocorpusculum* species appear to have a wider capacity for the utilisation of short-chain alcohols that was not observed in the host-associated strains. Previous analyses of the environmental species, *M. parvum* and *M. bavaricum*, demonstrated that they can utilise short-chain alcohols in CO_2_-dependent methanogenesis, with *M. parvum* able to use 2-propanol and 2-butanol [21]. Analysis of the respective *adh* further predicted the utilisation of cyclopentanol, 2,3-butanediol, ethanol, and 1-propanol for *M. parvum* and cyclopentanol and 2,3-butanediol for *M. bavaricum* [44]. The utilisation of short-chain alcohols by *M. parvum* was attributed to an *adh* (AKR1A1) that likely functions by converting 2-propanol and NADP to acetone and NADPH [45]. The absence of homologues of the *M. parvum adh* in all host-associated MAGs and isolate genomes suggests they are unable to utilise short-chain alcohols through this pathway. Indeed, we showed both *M. vombati* and *M. petauri* were unable to use short-chain alcohols as primary substrates for methanogenesis (Fig. 5). This simplified substrate profile is in contrast to *Methanosphaera*, which are found in the kangaroo gut and can utilise ethanol in methanol-dependent methanogenesis unlike other *Methanosphaera* spp. [12].

Contrary to the prevailing idea that *Methanocorpusculum* is primarily an environmental genus of methanogens, our expanded dataset indicates that this lineage was ancestrally host-associated and that one branch of the genus comprising the best characterised isolates (*M. parvum*, *M. labreanum*, and *M. bavaricum*) transitioned to an environmental lifestyle. Furthermore, the absence of alcohol-utilising *M. parvum adh* (AKR1A1) homologues in all host-associated lineages suggests the capacity to utilise short-chain alcohols through this pathway is a unique acquisition by the environmental *Methanocorpusculum* lineage (blue arrow in Fig. 3) and may indicate an adaptation to the hydrocarbon-rich environments in which these lineages are often found [17, 21]. However, *M. vombati*, HAC016, and HAC017 do contain homologues of uncharacterised archaeal and bacterial alcohol dehydrogenases, suggesting specific species of host-associated *Methanocorpusculum* may also have the capacity to utilise short-chain alcohols despite the absence of AKR1A1 though this requires validation.

Similarly, the host-associated *Methanocorpusculum* also contain a subset of genes indicative of host-specific adaptations. *M. vombati* is enriched for a cluster of genes characterised in the biosynthesis of phosphonate compounds such as the antibiotic dehydrophos produced by *Streptomyces luridus* and fosfomycin from *Streptomyces wedmorensis* [46–48], suggesting this lineage of *Methanocorpusculum* may also produce antibiotic phosphonate compounds to improve persistence within the gut. The ruminant *Methanocorpusculum* species contain a predicted bile salt hydrolase for the detoxification of bile acids [49], which is similarly encoded by the bovine-derived *Methanosphaera* sp. BMS [13]. In a recent analysis of bile acid metabolism in dairy cows, over one-third of MAGs analysed contained bile acid transformation pathways, including *Methanobrevibacter* and *Methanocorpusculum*, indicating the importance of bile acid metabolism as an adaptation to the bovine gastrointestinal tract [50]. Furthermore, the host-associated *Methanocorpusculum* contained a reduced capacity for the biosynthesis of tryptophan and other aromatic amino acids. This suggests the intestinal tract provides a constant source of exogenous amino acids to the host-associated species, where the inconsistent availability of amino acids to the environmental *Methanocorpusculum* has caused the need for biosynthesis.

## Conclusions

Through our characterisation of host-associated MAGs and isolate genomes, we have confirmed that the genus *Methanocorpusculum* is widely found in the gastrointestinal tract of herbivores and that each of the species encodes for unique genetic adaptations to their host environment. Furthermore, the ancestor of the *Methanocorpusculum* genus was likely host-associated and that these traits were lost in environmental lineage. Future studies are required to determine how these host-associated *Methanocorpusculum* species interact with the wider gut microbiome compared to other groups of methanogenic archaea, such as the typically dominant *Methanobrevibacter*.

### *Methanocorpusculum vombati* (sp. nov.)

*Methanocorpusculum* sp. CW153^T^ represents a novel species of *Methanocorpusculum* isolated from the Australian common wombat (*Vombatus ursinus*), for which we propose the name *Methanocorpusculum vombati* (sp. nov.; from *Vombatus*, denoting the genera of the common wombat form which it was isolated). This organism is a hydrogenotrophic methanogenic archaea, for which the type strain of the species is strain CW153^T^.

### *Methanocorpusculum petauri* (sp. nov.)

*Methanocorpusculum* sp. MG^T^ represents a novel species of *Methanocorpusculum* isolated from the Australian mahogany glider (*Petaurus gracilis*), for which we propose the name *Methanocorpusculum petauri* (sp. nov.; from *Petaurus*, denoting the genera of the mahogany glider form which it was isolated). This organism is a hydrogenotrophic methanogenic archaea, for which the type strain of the species is strain MG^T^.

## Methods

### Marsupial sample collection and storage

Marsupial faecal samples were collected from sanctuaries and zoos in South-East Queensland (Lone Pine Koala Sanctuary, Brisbane and David Fleay Wildlife Park, Burleigh Heads) and North Queensland (Wildlife Habitat, Port Douglas and Cairns Tropical Zoo). Faecal material was collected from 23 marsupial species, including greater glider (*n* = 4), mahogany glider (*n* = 9), squirrel glider (*n* = 4), yellow-bellied glider (*n* = 1), eastern grey kangaroo (*n* = 13), red kangaroo (*n* = 12), koala (*n* = 125), Lumholtz’s tree-kangaroo (*n* = 7), red-legged pademelon (*n* = 4), common brushtail possum (*n* = 10), common ringtail possum (*n* = 4), green ringtail possum (*n* = 5), Herbert River ringtail possum (*n* = 2), mountain brushtail possum (*n* = 1), short-eared possum (*n* = 3), striped possum (*n* = 2), agile wallaby (*n* = 3), northern nail-tail wallaby (n*=* 3 ), parma wallaby (*n* = 3), red-necked wallaby (*n* = 3), swamp wallaby (*n* = 2), common wombat (*n* = 6), and southern hairy-nosed wombat (*n* = 8). Ethical permission for the collection of all samples was granted by the Animal Welfare Unit, the University of Queensland, Brisbane, Australia, under ANRFA/SCMB/099/14. Half of each sample was stored at − 80 °C in Eppendorf tubes within 24 h and until processing. The other half was resuspended in RF30 [12, 51] and incubated at 37 °C for 24 h. Subsequently, ~ 2 mL of head space gas was retrieved for each tube using a sterile, gas-tight syringe and subjected to gas chromatography analyses, as described by Gagen et al. [52], using a Shimadzu GC-2014 (Shimadzu, Kyoto, Japan) fitted with a flame ionisation detector for CO_2_, H_2_, and CH_4_. A subsample of each culture was stored in anaerobic glycerol [53] at − 80 °C.

### Faecal sample DNA extraction, amplicon sequencing, and metagenome sequencing

A subset of collected marsupial faecal samples was chosen for DNA extraction (Additional file 1: Table S1). The faecal DNA extraction methods are described by Shiffman et al. [14]. For the 16S rRNA analyses, the V6–V8 hypervariable region (926F – 1392R [54];) of the 16S rRNA genes were amplified by PCR in 50 μL volumes containing 25 ng of DNA, 5 μL of 10× buffer, 1.5 μL of Bovine Serum Albumin (Roche diagnostic, Australia), 0.2 μL of 1 U Fisher Taq DNA polymerase (Thermo Fisher Scientific Inc., USA), 1 μL of dNTP mix (each at a concentration of 10 mM), 4 μL of 25 mM MgCl2, and 1 μL of each 10 mM of 926F and 1392R primers [54] ligated to Illumina adapter sequences. Each reaction was performed using the following cycling conditions: 95 °C for 3 min, followed by 30 cycles of 95 °C for 30 s, 55 °C for 30 s, 74 °C for 30 s, and a final extension at 74 °C for 10 min. AMPure XP beads (Beckman Coulter, Rea, CA, USA) were used to purify the resulting amplicons, as per the manufacturer’s instructions. Each sample was then indexed with unique 8-bp barcodes using the Illumina Nextera XT V2 Index Kit Set A-D (Illumina FC-131-1002; Illumina, San Diego, CA, USA) under standard PCR conditions. Equimolar indexed amplicons were pooled and sequenced at the Australian Centre for Ecogenomics, using the Illumina MiSeq platform with the version 3 reagent kit for 300 cycles, according to the manufacturer’s instructions. The raw data was demultiplexed and processed as per Shiffman et al. [14].

Four mahogany glider and four southern hairy-nosed wombat samples containing a high abundance of *Methanocorpusculum* were chosen for metagenomic sequencing. Aliquots of the extracted DNA were subjected to double-size selection for Illumina library preparation. First, 60 μL of AMPure XP beads (Beckman Coulter, Rea, CA, USA) was mixed with 100 μL of the DNA extract, vortexed, and held at room temperature for 5 min. The sample tubes were then placed on a magnetic stand for ~ 5 min, and once the solution was clear, the supernatant containing the desired DNA fragments was transferred to another sterile tube and the beads discarded. This process was repeated with 10 μL of AMPure XP beads (Beckman Coulter, Rea, CA, USA), and then the sample tube was placed on a magnetic as above, after which the supernatant was discarded. While remaining on the magnetic stand, the beads were washed by two rounds of exposure to 200 μL of 80% (v/v) ethanol for 30 s, with the ethanol removed at each step via pipette. The beads were then air-dried for ~ 15 min on the magnetic stand, and 25 μL of nuclease-free water was added, vortexed, and held at room temperature for 2 min. The mixtures were then placed on the magnetic stand for ~ 1 min (or until the solution was clear) and the liquid containing the eluted DNA was harvested via pipette and transferred to a new sterile tube. The DNA libraries for each sample were constructed using the Illumina Nextera XT DNA library preparation kit (Illumina, San Diego, CA, USA) and ~ 3 nM of each library was then sequenced using the Illumina NextSeq 500 platform with 2 × 150-bp paired-end chemistry, using standard protocols at the Australian Centre for Ecogenomics.

### Assessment of archaea prevalence and diversity in metagenomic sequencing datasets

A phylogenetic tree was constructed using the von Willebrand factor (vWF) of respective species available from the NCBI nucleotide database. The vWF genes were aligned using MUSCLE in MEGA-X [19], and phylogeny was inferred using maximum likelihood with 1000 bootstraps. The abundance of respective archaeal OTUs (above) was visualised against the marsupial phylogeny using pheatmap (v1.0.12) in RStudio (v2022.02.0-443).

### Recovery of archaeal MAGs from marsupial metagenomic sequencing datasets

SeqPurge with default settings (v.2018_11) [55] was used for adaptor trimming of the raw reads. Metaspades (v3.13.0) [56] was used to produce contiguous sequences, with auto PHRED offset and *k*-mer assembly lengths of 21, 33, and 55. BamM (v1.7.3; https://github.com/Ecogenomics/BamM) was used to map the paired-end reads of samples back to respective sample types (kangaroo, koala, glider, or wombat) and to produce contig coverage. UniteM (v0.0.16; https://github.com/dparks1134/UniteM) was used to recover MAGs from each sample, and CheckM (v1.0.12) [57] was used to assess quality. MAGs with ≥ 50% completeness and ≤ 10% contamination were retained and taxonomically assigned using GTDB-Tk (v1.0.2) [29]. The mean coverage of contigs for each MAG was determined using CoverM (v.0.4.0; https://github.com/wwood/CoverM) in contig mode.

### Methanogen enrichment and isolation from marsupial faecal samples

The wombat and mahogany glider samples that produced the highest CH_4_ for the given species were chosen for methanogen isolation. A 200-μL subsample of the wombat faecal slurry (CW153) was inoculated into 10 mL volumes of anaerobic BRN-RF10 medium [58] in Balch tubes and pressurised to 150 kPa with either H_2_:CO_2_ (80:20) or H_2_ gas. Cultures with H_2_ alone were supplemented with 1% (v/v) combinations of methanol (Sigma-Aldrich; 179337), ethanol (Sigma-Aldrich; E7023), 2-propanol (Sigma-Aldrich; I9516), 1-butanol (Sigma-Aldrich; 360465), 2 M sodium acetate, or 2 M trimethylamine (TMA) filter sterilised solutions. Streptomycin (600 μg/mL), ampicillin (200 μg/mL), and erythromycin (100 μg/mL) were added to all cultures, before incubation at 37 °C with rotational agitation at 100 rpm. Once subcultures of the enrichments were determined to be free of bacteria by PCR (27F/1492R) [59], CW153 cultures were then 10-fold serially diluted and 0.5 mL of the highest dilution which showed growth was transferred to BRN-RF10 (0.7%) agar roll tubes containing respective substrates. The roll tubes were incubated at 37 °C for 4–6 weeks. Random colonies were aseptically picked using a sterile glass Pasteur pipette and propagated in BRN-RF10 containing the respective gas/substrate combination.

For the mahogany glider enrichment, streptomycin and ampicillin were used but were changed for erythromycin (100 μg/mL) and vancomycin (50 μg/mL) after 10 subcultures to further suppress bacterial growth. One hundred microlitres of the bacteria-free enrichment cultures was spread on anaerobic BRN-RF10 agar (1.5% w/v), supplemented with respective substrates, in an anaerobic chamber (Coy Laboratory Products, MI, USA) with an atmosphere of CO_2_:H_2_:N_2_ (15:5:85). The agar plates were incubated at 37 °C for 4–6 weeks. Single colonies were picked from the agar and propagated in a broth medium as described above. The broth cultures from both rounds of enrichment were then evaluated for their purity and taxonomic origin using archaeal-specific 16S rRNA PCR (86F/1492R) [60]. PCR amplicons were cleaned using the Wizard SV Gel and PCR Clean-Up System gel extraction protocol, as per the manufacturer’s instructions, and sequenced at AGRF (https://www.agrf.org.au/). MEGA-X [19] was used to align the amplicon sequences with reference methanogen 16S rRNA sequences downloaded from the NCBI nucleotide database. Sequences were aligned using MUSCLE in MEGA-X, and phylogeny was inferred using maximum likelihood with 1000 bootstraps. The phylogenetic tree was visualised using iTOL (https://itol.embl.de/). Axenic broth cultures of the novel methanogens, hereafter referred to as *Methanocorpusculum* sp. CW153 and *Methanocorpusculum* sp. MG, were stored in anaerobic 30% glycerol solution at − 80 °C, prepared as per Teh et al. [53].

### Methanogen whole-genome sequencing

High-molecular weight genomic DNA was extracted from 10-mL cultures of each isolate using the consecutive freeze-thaw method described by Hoedt et al. [13], with 15 sets of consecutive freeze-thaws on dry ice for 5 min and 55 °C for 3 min. The quality and quantity of the genomic DNA samples were confirmed by Nanodrop and agarose gel electrophoresis, prior to genome sequencing at the Australian Centre for Ecogenomics. The Nextera DNA Flex Library Preparation Kit (Illumina #20018705) was used according to the manufacturer’s instructions, and the Mantis Liquid Handler (Formulatrix) was used for library preparation and cleanup. Each library was quality assessed using the TapeStation 4200 (Agilent #G2991AA) with Agilent D1000 HS tapes (#5067-5582) and quantified using the Quant-iT™ dsDNA HS Assay Kit (Invitrogen), as per the manufacturer’s instructions. Each library was sequenced using the Illumina NextSeq500 platform with NextSeq 500/550 High Output v2 2 × 150 bp paired-end chemistry and a sequencing depth of 1 Gbp for each sample.

Sequences were trimmed using Trimmomatic (v0.32) [61] and assembled using Spades (v3.14.1) [56]. The quality of each genome assembly was determined using CheckM (v1.1.2) [57], and taxonomic classification was performed using GTDB-Tk (v1.3.0) [29]. The coverage of each genome was determined using BamM (v1.7.3; https://github.com/Ecogenomics/BamM) and samtools mpileup. Predicted coding sequences were annotated using prokka (v1.14.6) [62] and the IMG Annotation Pipeline (v5.0.23; https://img.jgi.doe.gov/submit/) [63, 64]. BlastKOALA was used to assign KEGG Orthologs to the protein sequences of each genome [65].

### Microscopy and transmission electron microscopy of marsupial methanogen isolates

Methanogen isolates were cultured in BRN-RF10 medium with 150 kPa of CO_2_:H_2_ (20:80) headspace gas and respective substrates for methylotrophic strains. For light microscopy, samples of the cultures were heat-fixed on glass slides and stained using standard Gram staining protocols. Gram-stained slides were then imaged using a Nikon Eclipse 50i, under 100× magnification. Wet mount slides of each culture were visualised using a Zeiss AX10 epifluorescence microscope at 420 nm with a cyan (47 HE) filter set. Transmission electron microscopy of each isolate was conducted by Dr. Rick Webb at the University of Queensland Centre for Microscopy and Microanalysis (https://cmm.centre.uq.edu.au/). Cultures of each isolate were pelleted and mixed with low-gelling temperature agarose made with uninoculated BRN-RF10 medium. The sample was then immediately frozen using a Leica EMPACT2 high-pressure freezer. Each sample was then freeze-substituted (1% osmium tetroxide, 0.5% uranyl acetate, and 5% water in acetone), as per McDonald and Webb [66]. Samples were brought to room temperature and washed with acetone. Epon resin was used for infiltration and allowed to polymerise for 2 days at 60 °C. A Leica Ultracut UC6 ultramicrotome was used to produce ultrathin sections, which were picked up on Formvar-coated copper grids. The sections were stained with Reynolds lead citrate for 1 min and 5% uranyl acetate in 50% ethanol for 2 min and re-stained in Reynolds lead citrate again for 1 min, with a water wash after each subsequent step [67]. The sections were visualised, and micrographs were taken using a Hitachi HT7700 transmission electron microscope operated at 80 kV.

### Recovery of *Methanocorpusculum* MAGs from publicly available datasets

A total of 1276 metagenomes from 20 publicly available datasets were downloaded from the NCBI SRA database (https://www.ncbi.nlm.nih.gov/sra) between 18/05/2019 and 15/12/2020 (Additional file 1: Table S4). Each metagenome was trimmed using Trimmomatic (v0.32) [61] and assembled using MegaHit (v1.1.1) [68]. BamM (v1.7.3) was used to map the reads back to the assembly, and MetaBAT (v2.12.1) was used to produce genome bins with a minimum contig size of 1500 bps. Bin coverage was estimated using BamM and samtools as above. Bin quality was assessed using CheckM (v1.0.7) [57], and GTDB-Tk (v1.3.0) [29] was used to taxonomically assign each archaeal MAG. MAGs with ≥ 50% completeness and ≤ 10% were classified as medium-quality (MQ), and those with ≥ 90% completeness and ≤ 5% contamination were classified as high-quality (HQ). Predicted tRNAs and rRNAs were annotated using Aragorn and Barrnap within prokka (v1.14.6) [62], with the archaeal kingdom modifier. Multiple archaeal MAGs produced from a single metagenome were dereplicated using dRep (v2.4.0) [69].

### Phylogenetic analysis and average nucleotide identity of *Methanocorpusculum* genomes

Recovered MAGs, isolate genomes, and *Methanocorpusculum* downloaded from NCBI were taxonomically assigned and concatenated archaeal marker gene files were produced using GTDB-Tk (v1.6.0) [29]. Phylogeny was inferred using FastTree (v2.1.10) [30] and visualised by iToL (https://itol.embl.de/). The average nucleotide identity (ANI) was determined using fastani (v1.1) [70]. Average amino acid identity (AAI) was determined using EzAAI [71]. AAI was displayed as a heatmap using pheatmap (v1.0.12) in RStudio (v2022.02.0-443).

### Comparative analysis of *Methanocorpusculum* isolate genomes and MAGs

The HQ *Methanocorpusculum* MAGs and isolate genomes were included in the comparative genomic analyses. PCA plots of gene orthologs and KEGG Orthology variance were generated using EnrichM, with ‘--orthologs’ and ‘--ko’ annotation and subsequent enrichment functions (v0.4.15; https://github.com/geronimp/enrichM). Statistical analyses between the host groups were performed in EnrichM by Fisher’s exact test and Mann–Whitney *U* test. Corrected *p*-values of < 0.05 were considered significant. The percentage of genomes within host groups containing annotations was visualised using the pheatmaps (v1.0.12) package in RStudio (v2022.02.0-443).

### *Methanocorpusculum* growth kinetics and substrate utilisation

Growth curves for *M. petauri* MG and *M. vombati* CW153 were conducted in BRN-RF10 medium [58], without the addition of sodium acetate and sodium formate, and sparged using N_2_ gas. Ten millilitres of aliquots were prepared in Balch tubes and each pressurised with CO_2_, H_2_, or CO_2_/H_2_ (20:80) to 150 kPa. Cultures with CO_2_/H_2_ were used as positive controls and CO_2_ or H_2_ alone was used as the negative control. Substrate test cultures contained either CO_2_ or H_2_, along with 1% v/v supplementation of N_2_ sparged and filter sterilised substrates. Test substrates included methanol (Sigma-Aldrich; 179337), ethanol (Sigma-Aldrich; E7023), 1-propanol (Sigma-Aldrich; 402893), 2-propanol (Sigma-Aldrich; I9516), 1-butanol (Sigma-Aldrich; 360465), 2-butanol (Sigma-Aldrich; 19440), iso-butanol (Sigma-Aldrich; 320048), tert-butanol (Sigma-Aldrich; 360538), 1-pentanol (Sigma-Aldrich; 76929), 2-pentanol (Sigma-Aldrich; P8017), cyclopentanol (Sigma-Aldrich; C112208), cyclohexanol (Sigma-Aldrich; 105899), 2,3-butanediol (Sigma-Aldrich; B84904), or glycerol (Chem Supply; GA010). Sodium acetate (Sigma-Aldrich; S2289), sodium formate (Sigma-Aldrich; 798630), and methylamine (Sigma-Aldrich; M0505) were also included as 2-M solutions prepared in N_2_-sparged milli-Q H_2_O. Parent cultures were grown to the mid-exponential phase (0.2 OD_600_). Two hundred microlitres was aseptically inoculated into each tube in triplicate and incubated horizontally at 37 °C with 100 rpm rotational agitation. Growth was measured by OD_600_ at two-hourly intervals for ~ 36 h and then every ~ 24 h thereafter until ~ 500 h. Growth curves were visualised using GraphPad Prism 9.

## Supplementary Information

**Additional file 1: Table S1.** Methanogen abundance in marsupial samples by amplicon-based sequencing. **Table S2.** IMG JGI annotation of the *Methanocorpusculum petauri* MG and *Methanocorpusculum vombati* CW153 genomes. **Table S3.** Pairwise average nucleotide identity (ANI) of HQ *Methanocorpusculum*. NA denotes a pairwise ANI <70%. **Table S4.** List of publicly available metagenomes used to recover Methanocorpusculaceae MAGs. **Table S5.** Genome statistics for *Methanocorpusculum* MAG and isolate genomes included in this study. **Table S6.** List of genes significant enriched between Env and Host groups. **Table S7.** List of significantly enriched KOs between *Methanocorpusculum* host groups, as determined by Fisher’s Exact Test. **Table S8.** Proportions of genome group that contain the differentially enriched KOs.

**Additional file 2: Figure S1.** Hydrogen, methane, and carbon dioxide gas production by anaerobic cultures inoculated with marsupial faecal samples. **Figure S2.** Phylogenetic tree showing the preliminary taxonomic classification of marsupial methanogen enrichment cultures. **Figure S3.** Micrographs of *Methanocorpusculum* sp. CW153 and MG. **Figure S4.** Average amino acid identity (AAI) of high-quality *Methanocorpusculum* genomes and MAGs. **Figure S5.** Core and pan genome plots of host-associated and environmental *Methanocorpusculum* genomes. **Figure S6.** Core and pan genome plots of *Methanocorpusculum* species. **Figure S7.** Differential enrichment of genetic information processing genes in *Methanocorpusculum*. **Figure S8.** Host-specific enrichment of genes associated with the metabolism of cofactors and vitamins in *Methanocorpusculum*. **Figure S9.** Differential enrichment of amino acid metabolism genes in *Methanocorpusculum*. **Figure S10.** Differential enrichment of cell motility and defence genes in *Methanocorpusculum*. **Figure S11.** Differential enrichment of transport genes in *Methanocorpusculum*. **Figure S12.** Differential enrichment of other metabolism genes in *Methanocorpusculum*. **Figure S13.** Carbohydrate active enzymes (cazymes) annotated by *Methanocorpusculum* genomes. **Figure S14.** Substrate analysis of *M. petauri* in the presence of CO_2_.

## Data Availability

The datasets generated during this study are available on the NCBI database under the BioProject PRJNA907943 (https://www.ncbi.nlm.nih.gov/bioproject/PRJNA907943). Whole-genome sequences for *Methanocorpusculum vombati* CW153 and *Methanocorpusculum petauri* MG are available from GenBank under the accession number JAPTGC000000000 and JAPTGB000000000, respectively. All MAGs generated from this study are available from GenBank under the accession numbers JAQATW000000000 to JAQATX000000000, JAQAQY000000000 to JAQARF000000000, and DALTVW000000000 to DALUAV000000000. The shotgun metagenomic and 16S rRNA amplicon data have been submitted to the NCBI SRA database (https://www.ncbi.nlm.nih.gov/sra) under SRR22536466 to SRR22536474 and SRR22536364 to SRR22536465, respectively [72]. The publicly available datasets analysed during the current study are available from the NCBI SRA database [73–101].

## References

[1] Forster P, T. Storelvmo, K. Armour, W. Collins, J.-L. Dufresne, D. Frame, D.J. Lunt, T. Mauritsen, M.D. Palmer, M. Watanabe, M. Wild, and H. Zhang. The Earth’s energy budget, climate feedbacks, and climate sensitivity. Climate Change 2021: The Physical Science Basis Contribution of Working Group I to the Sixth Assessment Report of the Intergovernmental Panel on Climate Change. 2021: [Masson-Delmotte, V., P. Zhai, A. Pirani, S.L. Connors, C. Péan, S. Berger, N. Caud, Y. Chen, L. Goldfarb, M.I. Gomis, M. Huang, K. Leitzell, E. Lonnoy, J.B.R. Matthews, T.K. Maycock, T. Waterfield, O. Yelekçi, R. Yu, and B. Zhou (eds.)]. Cambridge University Press. In Press.

[2] IPCC. Climate Change 2021: The Physical Science Basis Contribution of Working Group I to the Sixth Assessment Report of the Intergovernmental Panel on Climate Change. 2021:[Masson-Delmotte, V., P. Zhai, A. Pirani, S. L. Connors, C. Péan, S. Berger, N. Caud, Y. Chen, L. Goldfarb, M. I. Gomis, M. Huang, K. Leitzell, E. Lonnoy, J.B.R. Matthews, T. K. Maycock, T. Waterfield, O. Yelekçi, R. Yu and B. Zhou (eds.)]. Cambridge University Press. In Press.

[3] Saunois M, Bousquet P, Poulter B, Peregon A, Ciais P, Canadell JG (2016). The global methane budget 2000–2012. Earth Syst Sci Data.

[4] Dangal SRS, Tian H, Zhang B, Pan S, Lu C, Yang J (2017). Methane emission from global livestock sector during 1890–2014: magnitude, trends and spatiotemporal patterns. Glob Chang Biol.

[5] Wolf J, Asrar GR, West TO (2017). Revised methane emissions factors and spatially distributed annual carbon fluxes for global livestock. Carbon Balance Manag.

[6] St-Pierre B, Wright AD (2013). Diversity of gut methanogens in herbivorous animals. Animal.

[7] Von Engelhardt W, Wolter S, Lawrenz H, Hemsley J (1978). Production of methane in two non-ruminant herbivores. Comp Biochem Physiol A Physiol.

[8] Vendl C, Clauss M, Stewart M, Leggett K, Hummel J, Kreuzer M (2015). Decreasing methane yield with increasing food intake keeps daily methane emissions constant in two foregut fermenting marsupials, the western grey kangaroo and red kangaroo. J Exp Biol.

[9] Ouwerkerk D, Maguire A, McMillen L, Klieve A (2009). Hydrogen utilising bacteria from the forestomach of eastern grey (Macropus giganteus) and red (Macropus rufus) kangaroos. Anim Prod Sci.

[10] Klieve AV, Ouwerkerk D, Maguire AJ (2012). Archaea in the foregut of macropod marsupials: PCR and amplicon sequence-based observations. J Appl Microbiol.

[11] Evans PN, Hinds LA, Sly LI, McSweeney CS, Morrison M, Wright A-DG (2009). Community composition and density of methanogens in the foregut of the tammar wallaby (Macropus eugenii). Appl Environ Microbiol.

[12] Hoedt EC, Cuív PÓ, Evans PN, Smith WJM, McSweeney CS, Denman SE (2016). Differences down-under: alcohol-fueled methanogenesis by archaea present in Australian macropodids. ISME J.

[13] Hoedt EC, Parks DH, Volmer JG, Rosewarne CP, Denman SE, McSweeney CS (2018). Culture- and metagenomics-enabled analyses of the Methanosphaera genus reveals their monophyletic origin and differentiation according to genome size. ISME J.

[14] Shiffman M, Soo R, Dennis P, Morrison M, Tyson G, Hugenholtz P (2017). Gene and genome-centric analyses of koala and wombat fecal microbiomes point to metabolic specialization for Eucalyptus digestion. PeerJ..

[15] Thomas CM, Desmond-Le Quéméner E, Gribaldo S, Borrel G (2022). Factors shaping the abundance and diversity of the gut archaeome across the animal kingdom. Nat Commun.

[16] Miller TL, Lin C (2002). Description of Methanobrevibacter gottschalkii sp. nov., Methanobrevibacter thaueri sp. nov., Methanobrevibacter woesei sp. nov. and Methanobrevibacter wolinii sp. nov. Int J Syst Evol Microbiol.

[17] Zhao Y, Boone DR, Mah RA, Boone JE, Xun L (1989). Isolation and characterization of Methanocorpusculum labreanum sp. nov. from the LaBrea Tar Pits. Int J Syst Evol Microbiol.

[18] Anderson IJ, Sieprawska-Lupa M, Goltsman E, Lapidus A, Copeland A, Glavina Del Rio T (2009). Complete genome sequence of Methanocorpusculum labreanum type strain Z. Stand Genomic Sci.

[19] Kumar S, Stecher G, Li M, Knyaz C, Tamura K (2018). MEGA X: molecular evolutionary genetics analysis across computing platforms. Mol Biol Evol.

[20] Richter M, Rosselló-Móra R (2009). Shifting the genomic gold standard for the prokaryotic species definition. Proc Natl Acad Sci U S A.

[21] Zellner G, Stackebrandt E, Messner P, Tindall BJ, de Conway ME, Kneifel H (1989). Methanocorpusculaceae fam. nov., represented by Methanocorpusculum parvum, Methanocorpusculum sinense spec. nov. and Methanocorpusculum bavaricum spec. nov. Arch Microbiol.

[22] Fernandes KA, Kittelmann S, Rogers CW, Gee EK, Bolwell CF, Bermingham EN (2014). Faecal microbiota of forage-fed horses in New Zealand and the population dynamics of microbial communities following dietary change. PLoS One.

[23] Graham DE, White RH (2002). Elucidation of methanogenic coenzyme biosyntheses: from spectroscopy to genomics. Nat Prod Rep.

[24] Cheeseman P, Toms-Wood A, Wolfe RS (1972). Isolation and properties of a fluorescent compound, factor 420, from Methanobacterium strain M.o.H. J Bacteriol.

[25] Nayfach S, Shi ZJ, Seshadri R, Pollard KS, Kyrpides NC (2019). New insights from uncultivated genomes of the global human gut microbiome. Nature..

[26] Parks DH, Rinke C, Chuvochina M, Chaumeil PA, Woodcroft BJ, Evans PN (2017). Recovery of nearly 8,000 metagenome-assembled genomes substantially expands the tree of life. Nat Microbiol.

[27] Xie F, Jin W, Si H, Yuan Y, Tao Y, Liu J (2021). An integrated gene catalog and over 10,000 metagenome-assembled genomes from the gastrointestinal microbiome of ruminants. Microbiome..

[28] Gilroy R, Ravi A, Getino M, Pursley I, Horton DL, Alikhan NF (2021). Extensive microbial diversity within the chicken gut microbiome revealed by metagenomics and culture. PeerJ..

[29] Chaumeil P-A, Mussig AJ, Hugenholtz P, Parks DH (2019). GTDB-Tk: a toolkit to classify genomes with the Genome Taxonomy Database. Bioinformatics.

[30] Price MN, Dehal PS, Arkin AP (2010). FastTree 2 – approximately maximum-likelihood trees for large alignments. PLoS One.

[31] Chaudhari NM, Gupta VK, Dutta C (2016). BPGA- an ultra-fast pan-genome analysis pipeline. Sci Rep.

[32] Gilmore SP, Henske JK, Sexton JA, Solomon KV, Seppala S, Yoo JI (2017). Genomic analysis of methanogenic archaea reveals a shift towards energy conservation. BMC Genomics.

[33] Médici R, Stammes H, Kwakernaak S, Otten LG, Hanefeld U (2017). Assessing the stereoselectivity of Serratia marcescens CECT 977 2,3-butanediol dehydrogenase. Catalysis Sci Technol.

[34] Kotani T, Yamamoto T, Yurimoto H, Sakai Y, Kato N (2003). Propane monooxygenase and NAD+-dependent secondary alcohol dehydrogenase in propane metabolism by Gordonia sp. Strain TY-5. J Bacteriol.

[35] Schweiger G, Dutscho R, Buckel W (1987). Purification of 2-hydroxyglutaryl-CoA dehydratase from Acidaminococcus fermentans. An iron-sulfur protein. Eur J Biochem.

[36] Porter AW, Young LY, Sariaslani S, Gadd GM (2014). Chapter five - Benzoyl-CoA, a universal biomarker for anaerobic degradation of aromatic compounds. Advances in Applied Microbiology.

[37] Najmudin S, Guerreiro CIPD, Carvalho AL, Prates JAM, Correia MAS, Alves VD (2006). Xyloglucan is recognized by carbohydrate-binding modules that interact with β-glucan chains*. J Biol Chem.

[38] Zellner G, Alten C, Stackebrandt E, de Conway ME, Winter J (1987). Isolation and characterization of Methanocorpusculum parvum, gen. nov., spec. nov., a new tungsten requiring, coccoid methanogen. Arch Microbiol.

[39] Hong P-Y, Wheeler E, Cann IKO, Mackie RI (2011). Phylogenetic analysis of the fecal microbial community in herbivorous land and marine iguanas of the Galápagos Islands using 16S rRNA-based pyrosequencing. ISME J.

[40] Joshi A, Lanjekar V, Dhakephalkar PK, Dagar SS (2018). Cultivation of multiple genera of hydrogenotrophic methanogens from different environmental niches. Anaerobe..

[41] Chibani CM, Mahnert A, Borrel G, Almeida A, Werner A, Brugère JF (2022). A catalogue of 1,167 genomes from the human gut archaeome. Nat Microbiol.

[42] Wright AD, Northwood KS, Obispo NE (2009). Rumen-like methanogens identified from the crop of the folivorous South American bird, the hoatzin (Opisthocomus hoazin). ISME J.

[43] García-González R, Aldezabal A, Laskurain NA, Margalida A, Novoa C (2016). Factors affecting diet variation in the Pyrenean rock ptarmigan (Lagopus muta pyrenaica): conservation implications. PLoS One.

[44] Bleicher K, Zellner G, Winter J (1989). Growth of methanogens on cyclopentanol/CO_2_ and specificity of alcohol dehydrogenase. FEMS Microbiol Lett.

[45] Bleicher K, Winter J (1991). Purification and properties of F420- and NADP(+)-dependent alcohol dehydrogenases of Methanogenium liminatans and Methanobacterium palustre, specific for secondary alcohols. Eur J Biochem.

[46] Shao Z, Blodgett JA, Circello BT, Eliot AC, Woodyer R, Li G (2008). Biosynthesis of 2-hydroxyethylphosphonate, an unexpected intermediate common to multiple phosphonate biosynthetic pathways. J Biol Chem.

[47] Nakashita H, Watanabe K, Hara O, Hidaka T, Seto H (1997). Studies on the biosynthesis of bialaphos. Biochemical mechanism of C-P bond formation: discovery of phosphonopyruvate decarboxylase which catalyzes the formation of phosphonoacetaldehyde from phosphonopyruvate. J Antibiot (Tokyo).

[48] Woodyer RD, Shao Z, Thomas PM, Kelleher NL, Blodgett JA, Metcalf WW (2006). Heterologous production of fosfomycin and identification of the minimal biosynthetic gene cluster. Chem Biol.

[49] Joyce SA, Gahan CG (2017). Disease-associated changes in bile acid profiles and links to altered gut microbiota. Dig Dis.

[50] Lin L, Lai Z, Yang H, Zhang J, Qi W, Xie F, Mao S. Genome-centric investigation of bile acid metabolizing microbiota of dairy cows and associated diet-induced functional implications. ISME J. 2023;17:172-184. 10.1038/s41396-022-01333-5 PMC9750977 36261508

[51] Joblin KN, Naylor GE, Williams AG (1990). Effect of Methanobrevibacter smithii on xylanolytic activity of anaerobic ruminal fungi. Appl Environ Microbiol.

[52] Gagen EJ, Wang J, Padmanabha J, Liu J, de Carvalho IP, Liu J (2014). Investigation of a new acetogen isolated from an enrichment of the tammar wallaby forestomach. BMC Microbiol.

[53] Teh JJ, Berendsen EM, Hoedt EC, Kang S, Zhang J, Zhang F, Liu Q, Hamilton AL, Wilson-O'Brien A, Ching J, Sung JJY, Yu J, Ng SC, Kamm MA, Morrison M. Novel strain-level resolution of Crohn’s disease mucosa-associated microbiota via an ex vivo combination of microbe culture and metagenomic sequencing. ISME J. 2021;15:3326–3338. 10.1038/s41396-021-00991-1 PMC8528831 34035441

[54] Engelbrektson A, Kunin V, Wrighton KC, Zvenigorodsky N, Chen F, Ochman H (2010). Experimental factors affecting PCR-based estimates of microbial species richness and evenness. ISME J.

[55] Sturm M, Schroeder C, Bauer P (2016). SeqPurge: highly-sensitive adapter trimming for paired-end NGS data. BMC Bioinformatics.

[56] Nurk S, Meleshko D, Korobeynikov A, Pevzner PA (2017). metaSPAdes: a new versatile metagenomic assembler. Genome Res.

[57] Parks DH, Imelfort M, Skennerton CT, Hugenholtz P, Tyson GW (2015). CheckM: assessing the quality of microbial genomes recovered from isolates, single cells, and metagenomes. Genome Res.

[58] Balch WE, Fox GE, Magrum LJ, Woese CR, Wolfe RS (1979). Methanogens: reevaluation of a unique biological group. Microbiol Rev.

[59] Enticknap JJ, Kelly M, Peraud O, Hill RT (2006). Characterization of a culturable alphaproteobacterial symbiont common to many marine sponges and evidence for vertical transmission via sponge larvae. Appl Environ Microbiol.

[60] Wright AD, Pimm C (2003). Improved strategy for presumptive identification of methanogens using 16S riboprinting. J Microbiol Methods.

[61] Bolger AM, Lohse M, Usadel B (2014). Trimmomatic: a flexible trimmer for Illumina sequence data. Bioinformatics.

[62] Seemann T (2014). Prokka: rapid prokaryotic genome annotation. Bioinformatics.

[63] Chen IA, Chu K, Palaniappan K, Pillay M, Ratner A, Huang J (2019). IMG/M v.5.0: an integrated data management and comparative analysis system for microbial genomes and microbiomes. Nucleic Acids Res.

[64] Huntemann M, Ivanova NN, Mavromatis K, Tripp HJ, Paez-Espino D, Palaniappan K (2015). The standard operating procedure of the DOE-JGI Microbial Genome Annotation Pipeline (MGAP v.4). Stand Genomic Sci.

[65] Kanehisa M, Sato Y, Morishima K (2016). BlastKOALA and GhostKOALA: KEGG tools for functional characterization of genome and metagenome sequences. J Mol Biol.

[66] McDonald KL, Webb RI (2011). Freeze substitution in 3 hours or less. J Microsc.

[67] Daddow LYM (1983). A double lead stain method for enhancing contrast of ultrathin sections in electron microscopy: a modified multiple staining technique. J Microsc.

[68] Li D, Liu CM, Luo R, Sadakane K, Lam TW (2015). MEGAHIT: an ultra-fast single-node solution for large and complex metagenomics assembly via succinct de Bruijn graph. Bioinformatics.

[69] Olm MR, Brown CT, Brooks B, Banfield JF (2017). dRep: a tool for fast and accurate genomic comparisons that enables improved genome recovery from metagenomes through de-replication. ISME J.

[70] Jain C, Rodriguez-R LM, Phillippy AM, Konstantinidis KT, Aluru S (2018). High throughput ANI analysis of 90K prokaryotic genomes reveals clear species boundaries. Nat Commun.

[71] Kim D, Park S, Chun J (2021). Introducing EzAAI: a pipeline for high throughput calculations of prokaryotic average amino acid identity. J Microbiol.

[72] Volmer JG, Soo RM, Evans PN, Hoedt EC, Astorga Alsina AL, Woodcroft BJ, Tyson GW, Hugenholtz P, Morrison M. Characterisation and isolation of novel host-associated Methanocorpusculum species from native Australian herbivores. NCBI SRA Database. 2022. https://www.ncbi.nlm.nih.gov/Traces/study/?page=3&acc=SRP411434. 10.1186/s12915-023-01524-2 PMC10035134 36949471

[73] Gilroy R, Leng J, Ravi A, Adriaenssens EM, Oren A, Baker D (2022). Metagenomic investigation of the equine faecal microbiome reveals extensive taxonomic diversity. PeerJ..

[74] Gilroy R LJ, Ravi A, Adriaenssens EM, Oren A, Baker D, La Ragione RM, Proudman C, Pallen MJ. Shotgun metagenome of horse feces. NCBI SRA Database. 2019. https://www.ncbi.nlm.nih.gov/Traces/study/?acc=SRP230873.

[75] Gut metagenomic data of young juvenile female Asian elephant (Elephas maximus). NCBI SRA Database. 2019. https://www.ncbi.nlm.nih.gov/Traces/study/?acc=SRP200208&o=acc_s%3Aa.

[76] Comprehensive metagenomic. NCBI SRA Database. 2017. https://www.ncbi.nlm.nih.gov/Traces/study/?acc=SRP127637.

[77] Gut metagenomic data of young juvenile male Asian elephant (Elephas maximus). NCBI SRA Database. 2019. https://www.ncbi.nlm.nih.gov/Traces/study/?acc=SRP200206.

[78] Gut metagenomic data of old adult male Asian elephant (Elephas maximus). NCBI SRA Database. 2019. https://www.ncbi.nlm.nih.gov/Traces/study/?acc=SRP200212.

[79] Cao J, Hu Y, Liu F, Wang Y, Bi Y, Lv N, Li J, Zhu B, Gao GF. bird metagenome Raw sequence reads. NCBI SRA Database. 2019. https://www.ncbi.nlm.nih.gov/Traces/study/?acc=SRP216618.

[80] Cao J, Hu Y, Liu F, Wang Y, Bi Y, Lv N, Li J, Zhu B, Gao GF. Metagenome sequencing Raw sequence reads. NCBI SRA Database. 2019. https://www.ncbi.nlm.nih.gov/Traces/study/?acc=SRP220891.

[81] Doster E, Rovira P, Noyes NR, Burgess BA, Yang X, Weinroth MD, Lakin SM, Dean CJ, Linke L, Magnuson R, Jones KI, Boucher C, Ruiz J, Belk KE, Morley PS. Cattle feces metagenome. NCBI SRA Database. 2016. https://www.ncbi.nlm.nih.gov/Traces/study/?acc=SRP069825.

[82] Gibson KM, Nguyen BN, Neumann LM, Miller M, Buss P, Daniels S (2019). Gut microbiome differences between wild and captive black rhinoceros – implications for rhino health. Sci Rep.

[83] Gibson KM, Nguyen BN, Neumann LM, Miller M, Buss P, Daniels S, et al. Gut microbiome differences between wild and captive black rhinoceros – implications for rhino health. NCBI SRA Database. 2019. https://www.ncbi.nlm.nih.gov/Traces/study/?acc=SRP192412. 10.1038/s41598-019-43875-3 PMC6538756 31138833

[84] Hou Q, Kwok LY, Zheng Y, Wang L, Guo Z, Zhang J, Huang W, Wang Y, Leng L, Li H, Zhang H. Differential fecal microbiota are retained in broiler chicken lines divergently selected for fatness traits. NCBI SRA Database. 2016. https://www.ncbi.nlm.nih.gov/Traces/study/?acc=PRJNA340908. 10.1038/srep37376 PMC5120256 27876778

[85] Hou Q, Kwok LY, Zheng Y, Wang L, Guo Z, Zhang J, Huang W, Wang Y, Leng L, Li H, Zhang H. Differential fecal microbiota are retained in broiler chicken lines divergently selected for fatness traits. Sci Rep. 2016;6(1):37376. 10.1038/srep37376 PMC5120256 27876778

[86] Ilmberger N, Güllert S, Dannenberg J, Rabausch U, Torres J, Wemheuer B, Alawi M, Poehlein A, Chow J, Turaev D, Rattei T, Schmeisser C, Salomon J, Olsen PB, Daniel R, Grundhoff A, Borchert MS, Streit WR. Elephant feces metagenome. NCBI SRA Database. 2014. https://www.ncbi.nlm.nih.gov/Traces/study/?acc=PRJNA240141.

[87] Ilmberger N, Güllert S, Dannenberg J, Rabausch U, Torres J, Wemheuer B (2014). A comparative metagenome survey of the fecal microbiota of a breast- and a plant-fed Asian elephant reveals an unexpectedly high diversity of glycoside hydrolase family enzymes. PLoS One.

[88] Li C, Tan X, Bai J, Xu Q, Liu S, Guo W, Yu C, Fan G, Lu Y, Zhang H, Yang H, Chen J, Liu X. Sperm whale genome sequencing and assembly. NCBI SRA Database. 2017. https://www.ncbi.nlm.nih.gov/Traces/study/?acc=PRJNA411766.

[89] Li C, Tan X, Bai J, Xu Q, Liu S, Guo W (2019). A survey of the sperm whale (Physeter catodon) commensal microbiome. PeerJ..

[90] Suk-Kyung Lim DK, Dong-Chan Moon, Youna Cho, Mina Rho. Whole metagenomic sequencing for animal (pig&cattle) gut microbiome. GigaScience. 2019. 10.1093/gigascience/giaa043.

[91] Controlling enteric pathogens of poultry gut. NCBI SRA Database. 2019. https://www.ncbi.nlm.nih.gov/Traces/study/?acc=PRJEB23356.

[92] Chicken, pig and cattle gut microbiome raw sequence reads. NCBI SRA Database. 2015. https://www.ncbi.nlm.nih.gov/Traces/study/?acc=PRJNA293646.

[93] Salgado-Flores A, Tveit AT, Wright AD, Pope PB, Sundset MA. Ptarmigans cecum microbiome. NCBI SRA Database. 2018. https://www.ncbi.nlm.nih.gov/Traces/study/?acc=PRJNA450906. 10.1371/journal.pone.0213503 PMC6411164 30856229

[94] Salgado-Flores A, Tveit AT, Wright A-D, Pope PB, Sundset MA (2019). Characterization of the cecum microbiome from wild and captive rock ptarmigans indigenous to Arctic Norway. PLoS One.

[95] Wang H, Yan Y, Yi X, Duan Y, Wang J, Li S, Luo L, Huang T, Inglis B, Li X, Ji W, Tan T, Si W. Gut microbiota of rhesus monkey. NCBI SRA Database. 2018. https://www.ncbi.nlm.nih.gov/Traces/study/?acc=PRJNA483083.

[96] Wang H, Yan Y, Yi X, Duan Y, Wang J, Li S (2019). Histopathological features and composition of gut microbiota in rhesus monkey of alcoholic liver disease. Front Microbiol.

[97] Zaheer R, Lakin SM, Polo RO, Cook SR, Larney FJ, Morley PS, Booker CW, Hannon SJ, Van Domselaar G, Read RR, McAllister TA. Bovine fecal metagenome. NCBI SRA Database. 2017. https://www.ncbi.nlm.nih.gov/Traces/study/?acc=PRJNA420682.

[98] Zaheer R, Lakin SM, Polo RO, Cook SR, Larney FJ, Morley PS (2019). Comparative diversity of microbiomes and Resistomes in beef feedlots, downstream environments and urban sewage influent. BMC Microbiol.

[99] Zaheer R, Lakin SM, Polo RO, Cook SR, Larney FJ, Morley PS, et al. Bovine feedlot catch-basin metagenome. NCBI SRA Database. 2019. https://www.ncbi.nlm.nih.gov/Traces/study/?acc=PRJNA529711.

[100] Rovira Sanz P. Impact of antibiotic use on resistance in beef feedlot and dairy cattle: Colorado State University; 2017.

[101] Rovira Sanz P. Farm metagenomes conventional and organic. NCBI SRA Database. 2017. https://www.ncbi.nlm.nih.gov/Traces/study/?acc=PRJNA379303.

