# Supplementary material for: Isolation and characterisation of novel Methanocorpusculum species indicates the genus is ancestrally host-associated
Source: BMC Biol. 2023 Mar 22;21:59. doi: 10.1186/s12915-023-01524-2 (PMC10035134; doi:10.1186/s12915-023-01524-2)
Supplement: Supplementary file 2 — Additional file 2: Figure S1. Hydrogen, methane, and carbon dioxide gas production by anaerobic cultures inoculated with marsupial faecal samples. Figure S2. Phylogenetic tree showing the preliminary taxonomic classification of marsupial methanogen enrichment cultures. Figure S3. Micrographs of Methanocorpusculum sp. CW153 and MG. Figure S4. Average amino acid identity (AAI) of high-quality Methanocorpusculum genomes and MAGs. Figure S5. Core and pan genome plots of host-associated and environmental Methanocorpusculum genomes. Figure S6. Core and pan genome plots of Methanocorpusculum species. Figure S7. Differential enrichment of genetic information processing genes in Methanocorpusculum. Figure S8. Host-specific enrichment of genes associated with the metabolism of cofactors and vitamins in Methanocorpusculum. Figure S9. Differential enrichment of amino acid metabolism genes in Methanocorpusculum. Figure S10. Differential enrichment of cell motility and defence genes in Methanocorpusculum. Figure S11. Differential enrichment of transport genes in Methanocorpusculum. Figure S12. Differential enrichment of other metabolism genes in Methanocorpusculum. Figure S13. Carbohydrate active enzymes (cazymes) annotated by Methanocorpusculum genomes. Figure S14. Substrate analysis of M. petauri in the presence of CO2. [file 12915_2023_1524_MOESM2_ESM.pdf]

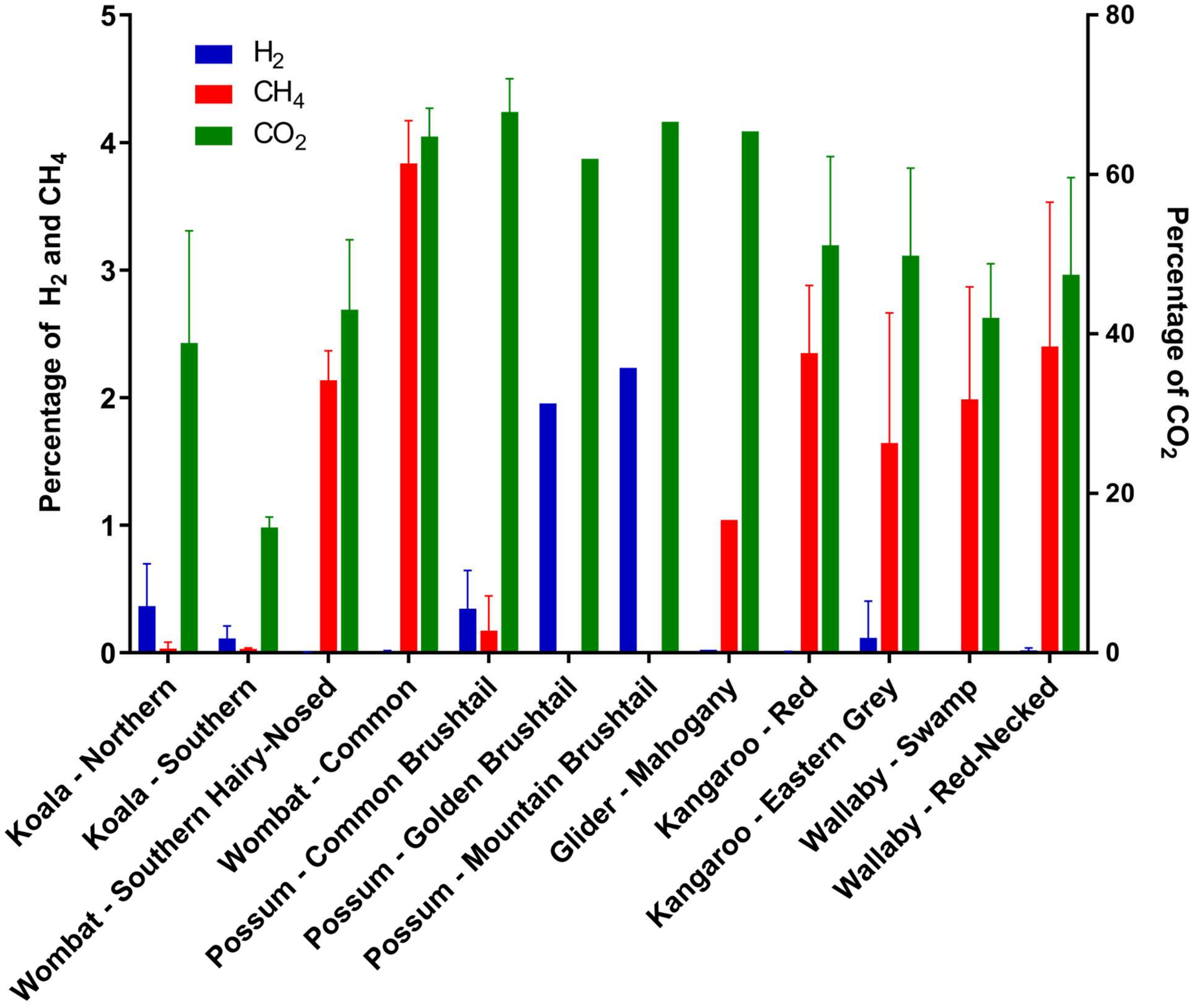

Figure S1. Hydrogen, methane, and carbon dioxide gas production by anaerobic cultures inoculated with marsupial faecal samples. Gas production was analysed after 24h incubation using gas chromatography. Values represent average percentages of culture headspace gas samples for the given species. Samples from Koala (Northern n=109, Southern n=2), Southern hairy-nosed wombat (n=5), common wombat (n=3), common brushtail possum (common n=3, golden n=1), mountain brushtail possum (n=1), mahogany glider (n=1), red kangaroo (n=12), Eastern grey kangaroo (n=10), swamp wallaby (n=2), and red-necked wallaby (n=3) were included.

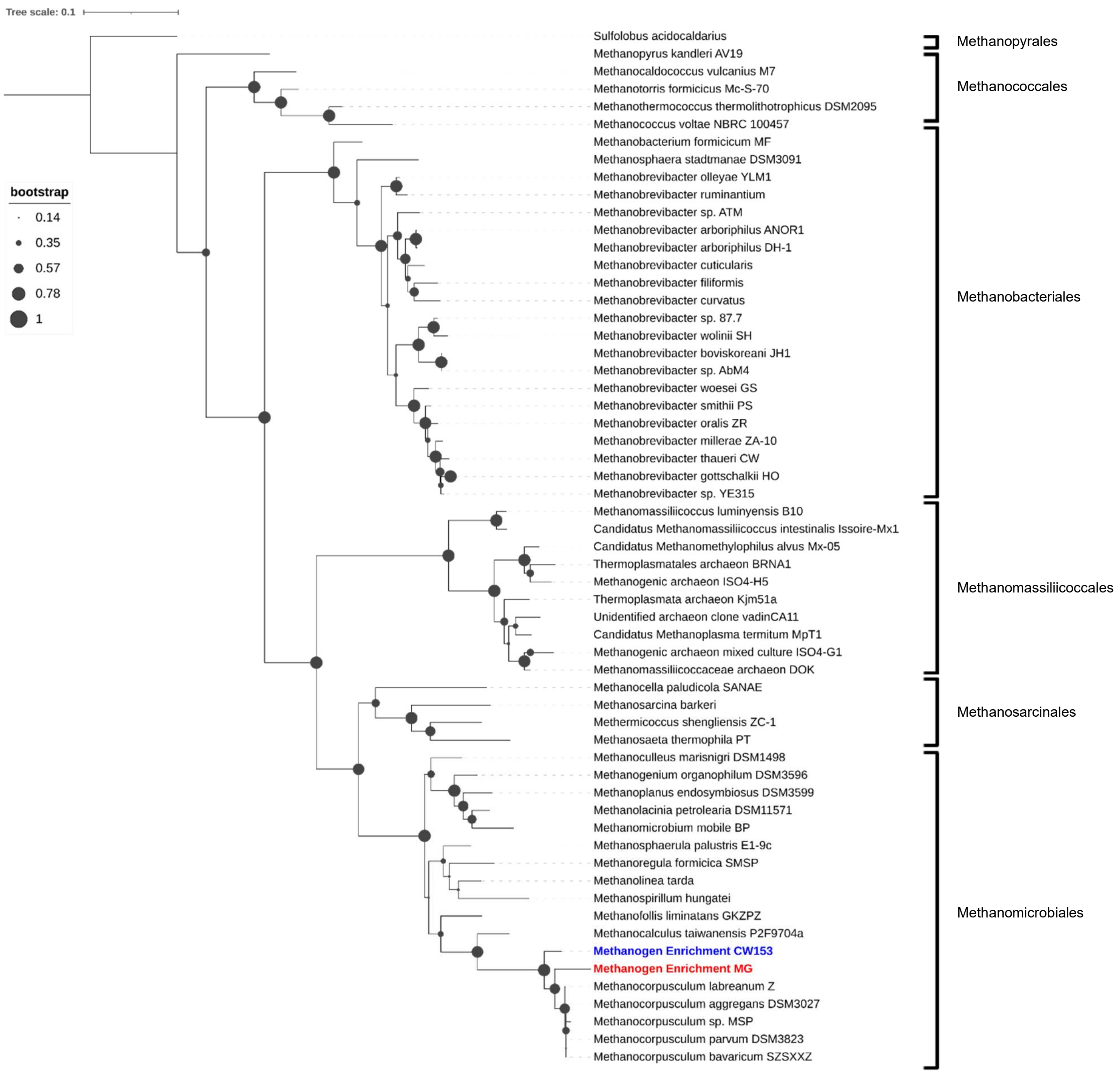

Figure S2. Phylogenetic tree showing the preliminary taxonomic classification of marsupial methanogen enrichment cultures. 16S rRNA amplicons were generated from respective isolates using the 86F/1392R archaeal primers. MEGA-X (4) was then used to align the amplicon sequences with reference methanogen 16S rRNA sequences downloaded from the NCBI nucleotide database. Sequences were aligned using MUSCLE, and phylogeny inferred using Maximum-likelihood and 1000 bootstraps. The mahogany glider and wombat enrichments are displayed in red and blue, respectively. The scale bar represents 10% sequence divergence and *Sulfolobus acidocaldarius* was used as the outgroup.

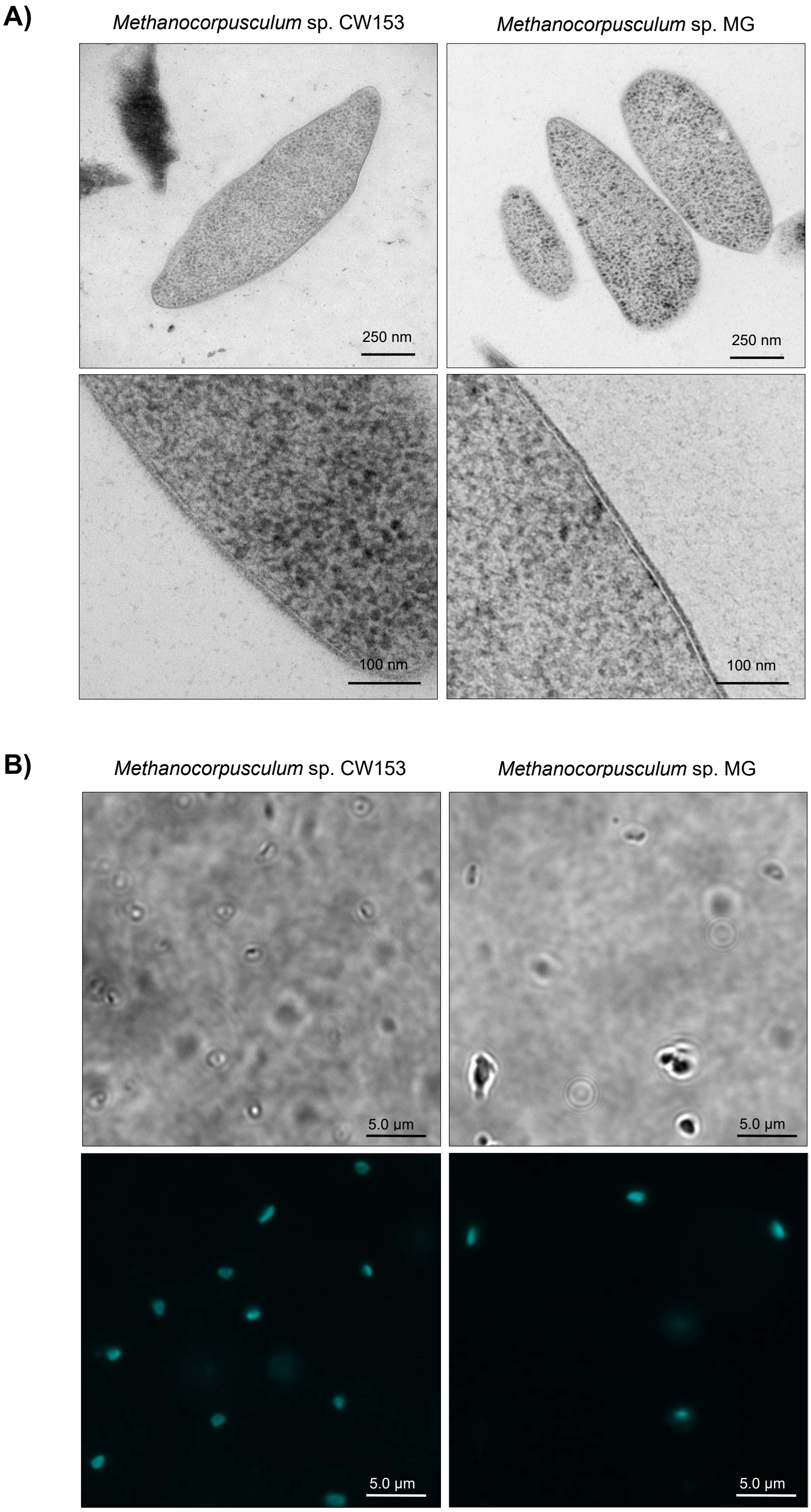

Figure S3. Micrographs of *Methanocorpusculum* sp. CW153 and MG. A) Transmission electron micrographs (TEM) of *Methanocorpusculum* sp. CW153 and MG. **B)** Phase contrast and epifluorescence (420 nm with a cyan 47 HE filter set) micrographs of *Methanocorpusculum* sp. CW153 and MG.

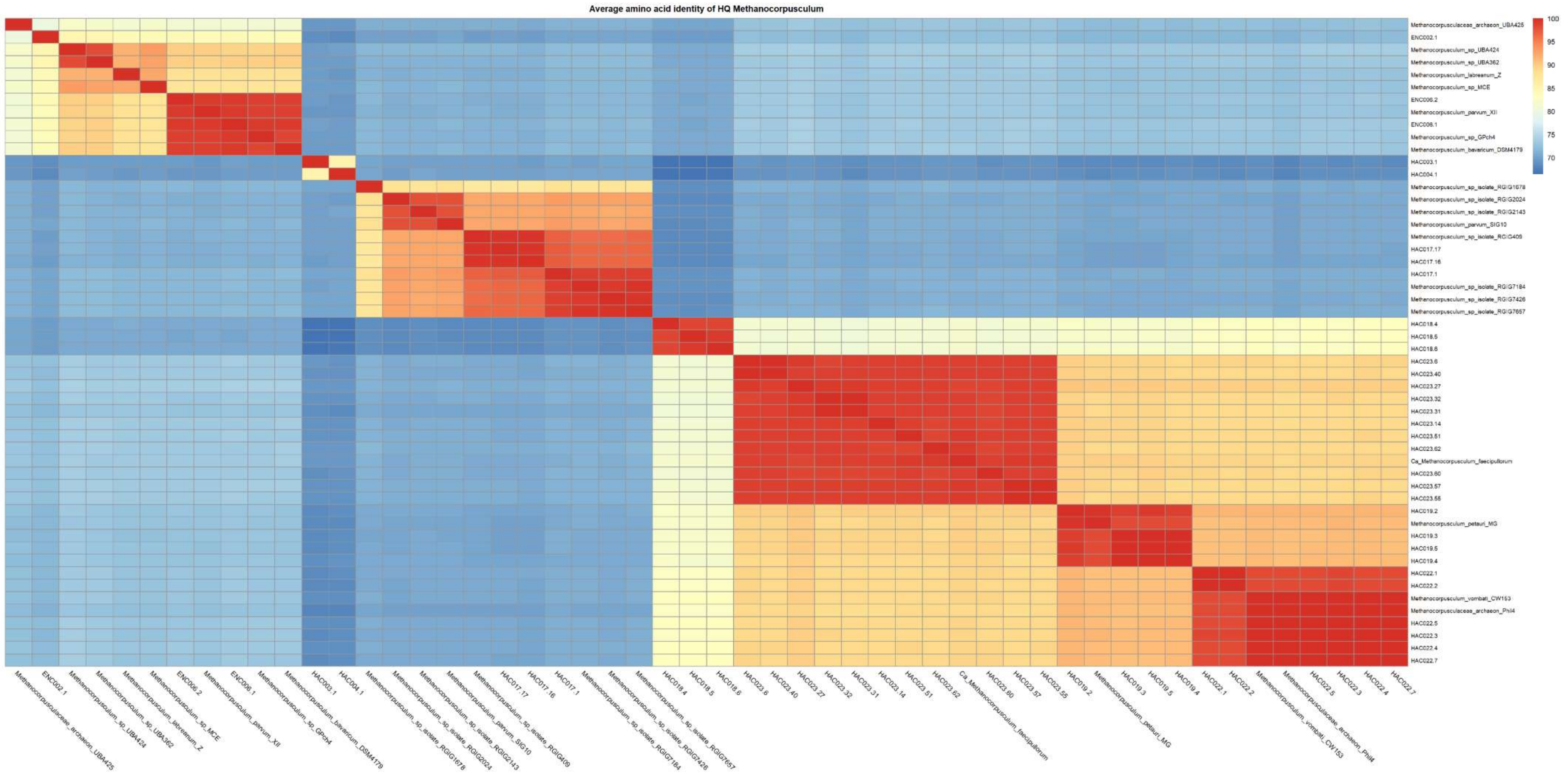

Figure S4. Average amino acid identity (AAI) of high-quality *Methanocorpusculum* genomes and MAGs. The AAI matrix of HQ genomes was generated using EzAAI (1) and visualised using the pheatmaps (v1.0.12) package in R studio. Percentage AAI identity between respective genomes is colour coded according to the legend.

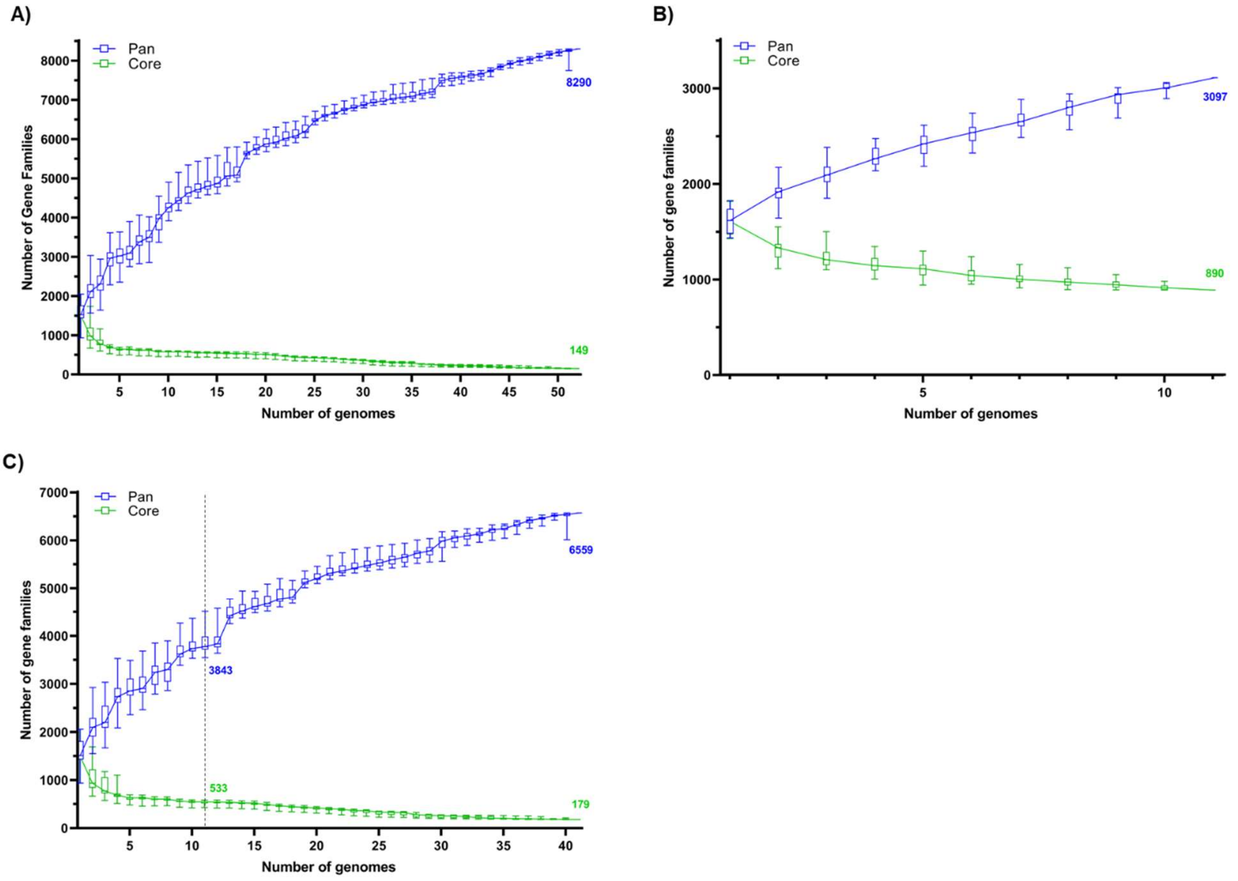

Figure S5. Core and pan genome plots of host-associated and environmental *Methanocorpusculum* genomes. Core and pan genome analysis was conducted using the Bacterial Pan Genome Analysis software (2), with 100 randomised replications. **A)** All *Methanocorpusculum*, **B)** environmental *Methanocorpusculum*, **C)** host-associated *Methanocorpusculum*. For **C)**, values for the core and pan genome are shown at 41 and 11 genomes, as only 11 Env genomes are available.

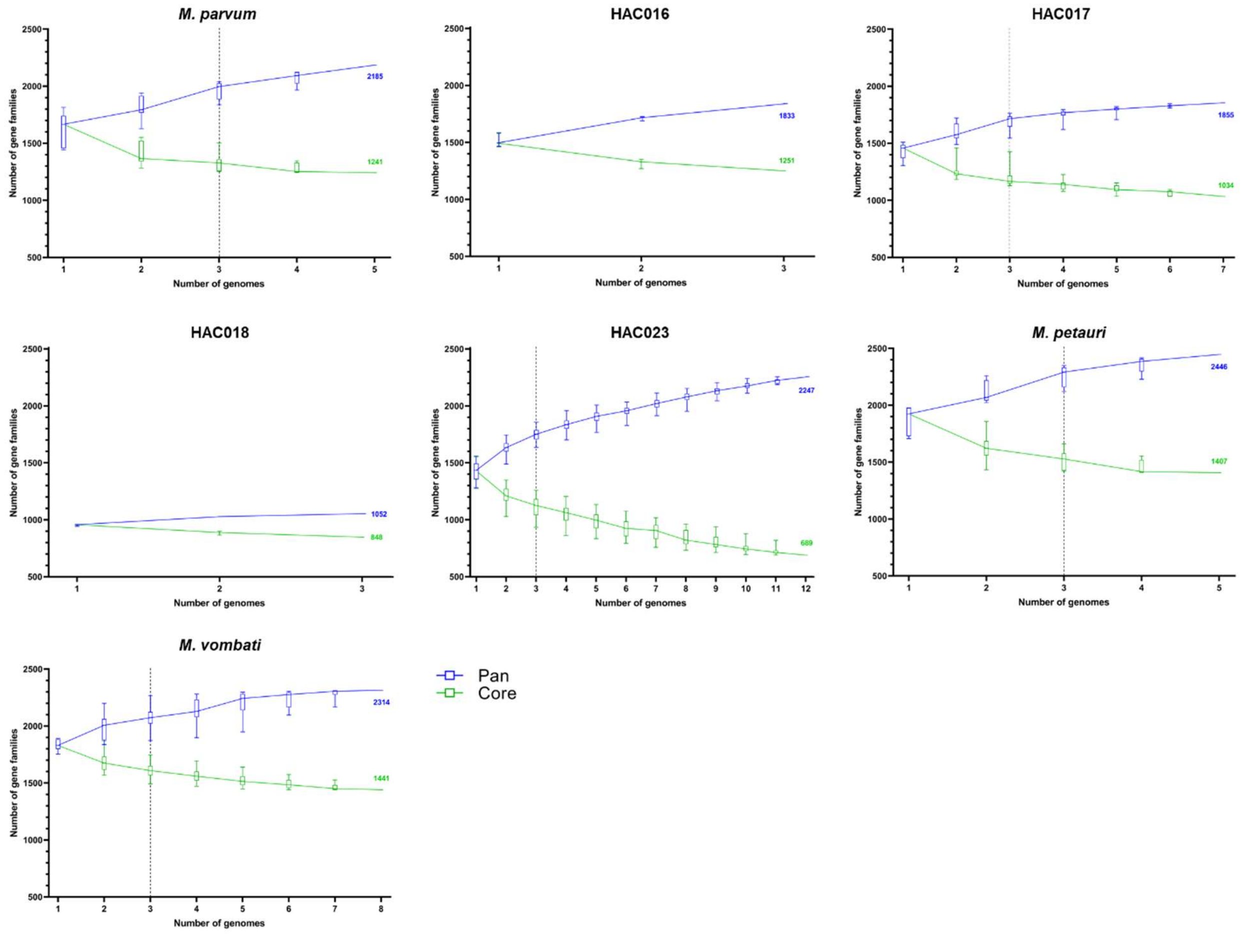

Figure S6. Core and pan genome plots of *Methanocorpusculum* species. Core and pan genome analysis was conducted using the Bacterial Pan Genome Analysis software (2), with 100 random replications. Species with ≥ three representative genomes were included. As such, sp002506085, ENC002, sp002498375, *M. labreanum*, sp003315675, HAC003, HAC004, and HAC013 were excluded from the analysis.

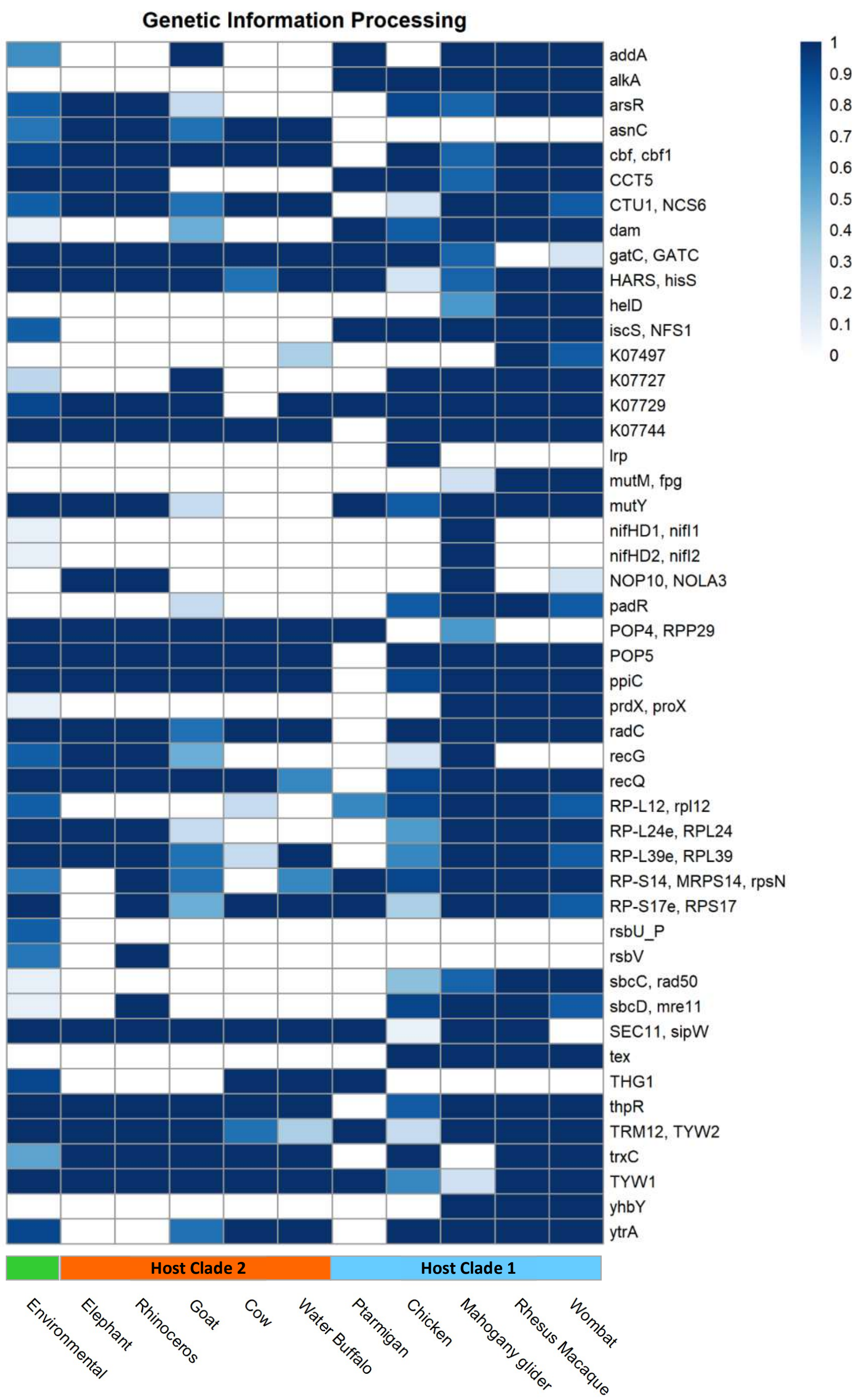

Figure S7. Differential enrichment of genetic information processing genes in *Methanocorpusculum*. KO annotation and statistical analyses were performed using the ‘annotate’ and ‘enrichment’ functions of EnrichM (v0.4.15; https://github.com/geronimp/enrichM). Genomes were grouped by host species and compared by Fisher’s Exact Test, where KOs with corrected p values of <0.05 were retained and considered significant. Heatmap values are colour coded according to the legend and represent the proportion of respective genomes for a given host group. The *Methanocorpusculum* are also labelled as Environmental Clade (green), Host Clade 2 (orange), and Host Clade 1 (blue), as per Figure 3.

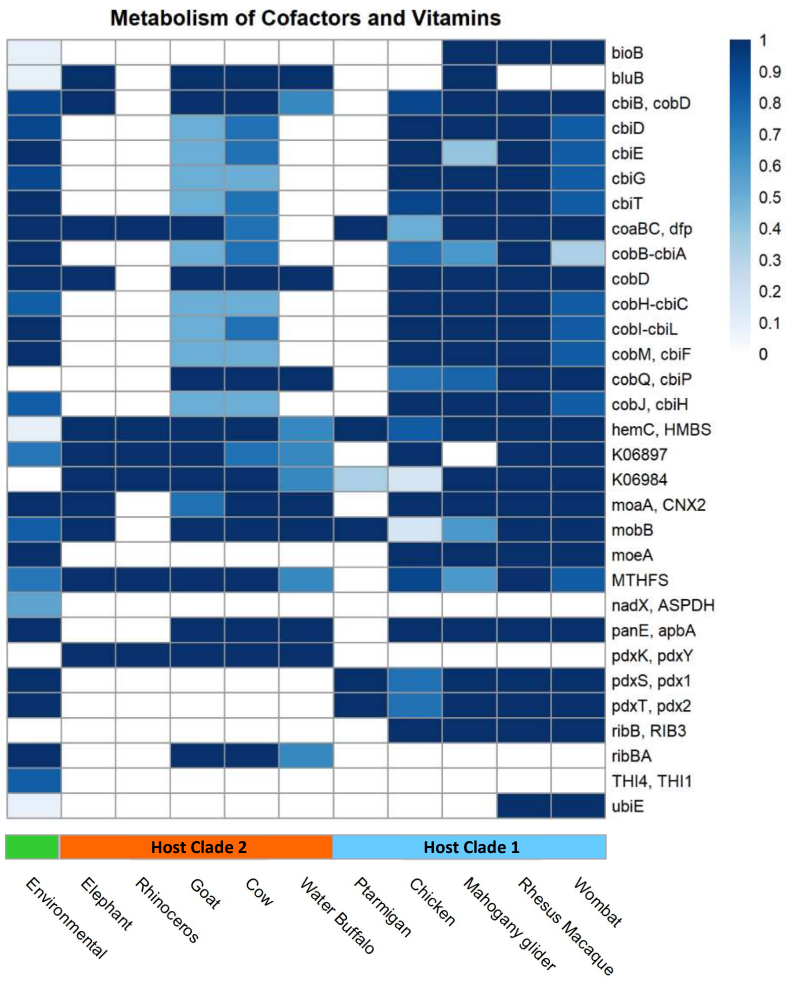

Figure S8. Host-specific enrichment of genes associated with the metabolism of cofactors and vitamins in *Methanocorpusculum*. KO annotation and statistical analyses were performed using the ‘annotate’ and ‘enrichment’ functions of EnrichM (v0.4.15; https://github.com/geronimp/enrichM). Genomes were grouped by host species and compared by Fisher’s Exact Test, where KOs with corrected p values of <0.05 were retained and considered significant. Heatmap values are colour coded according to the legend and represent the proportion of respective genomes for a given host group. The *Methanocorpusculum* are also labelled as Environmental Clade (green), Host Clade 2 (orange), and Host Clade 1 (blue), as per Figure 3.

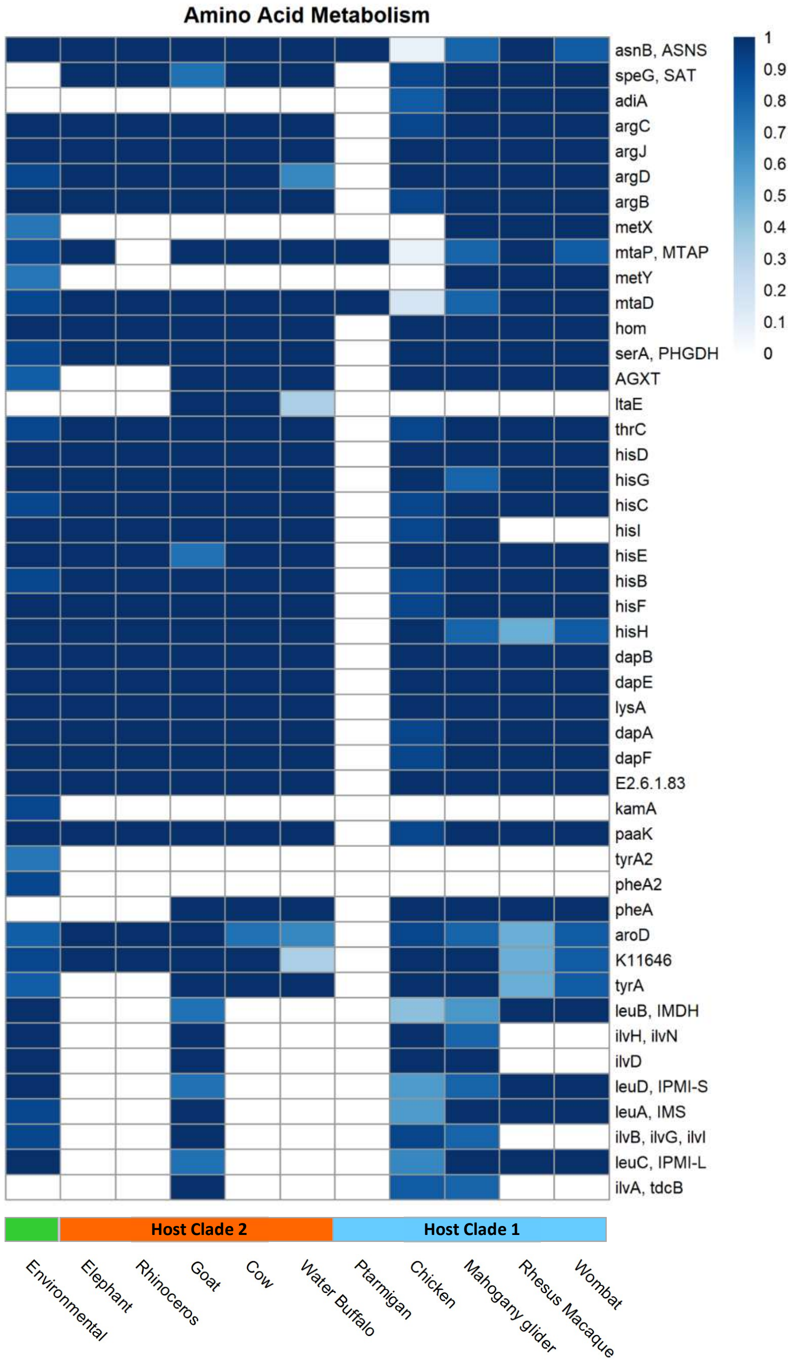

Figure S9. Differential enrichment of amino acid metabolism genes in *Methanocorpusculum*. KO annotation and statistical analyses were performed using the 'annotate' and 'enrichment' functions of EnrichM (v0.4.15; https://github.com/geronimp/enrichM). Genomes were grouped by host species and compared by Fisher's Exact Test, where KOs with corrected p values of <0.05 were retained and considered significant. Heatmap values are colour coded according to the legend and represent the proportion of respective genomes for a given host group. The *Methanocorpusculum* are also labelled as Environmental Clade (green), Host Clade 2 (orange), and Host Clade 1 (blue), as per Figure 3.

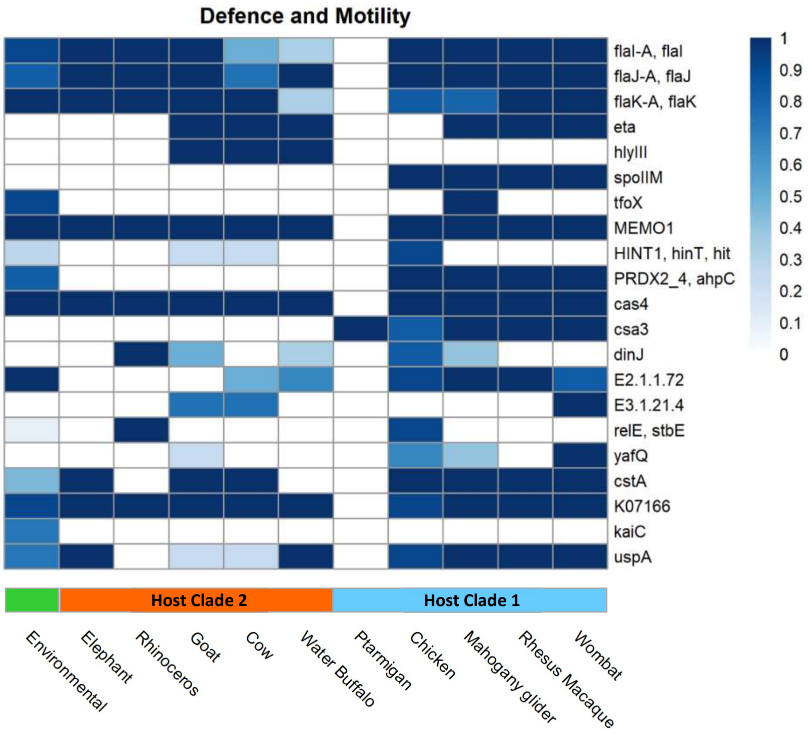

Figure S10. Differential enrichment of cell motility and defence genes in *Methanocorpusculum*. KO annotation and statistical analyses were performed using the 'annotate' and 'enrichment' functions of EnrichM (v0.4.15; https://github.com/geronimp/enrichM). Genomes were grouped by host species and compared by Fisher's Exact Test, where KOs with corrected p values of <0.05 were retained and considered significant. Heatmap values are colour coded according to the legend and represent the proportion of respective genomes for a given host group. The *Methanocorpusculum* are also labelled as Environmental Clade (green), Host Clade 2 (orange), and Host Clade 1 (blue), as per Figure 3.

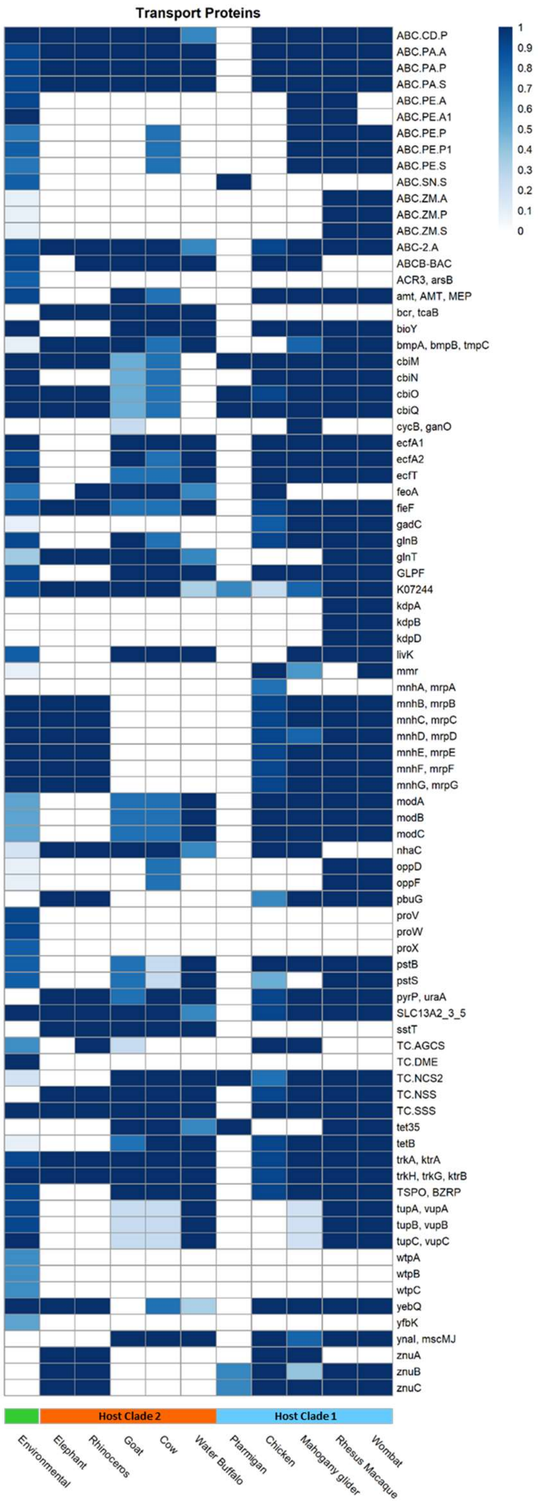

Figure S11. Differential enrichment of transport genes in *Methanocorpusculum*. KO annotation and statistical analyses were performed using the 'annotate' and 'enrichment' functions of EnrichM (v0.4.15; https://github.com/geronimp/enrichM). Genomes were grouped by host species and compared by Fisher's Exact Test, where KOs with corrected p values of <0.05 were retained and considered significant. Heatmap values are colour coded according to the legend and represent the proportion of respective genomes for a given host group. The *Methanocorpusculum* are also labelled as Environmental Clade (green), Host Clade 2 (orange), and Host Clade 1 (blue), as per Figure 3.

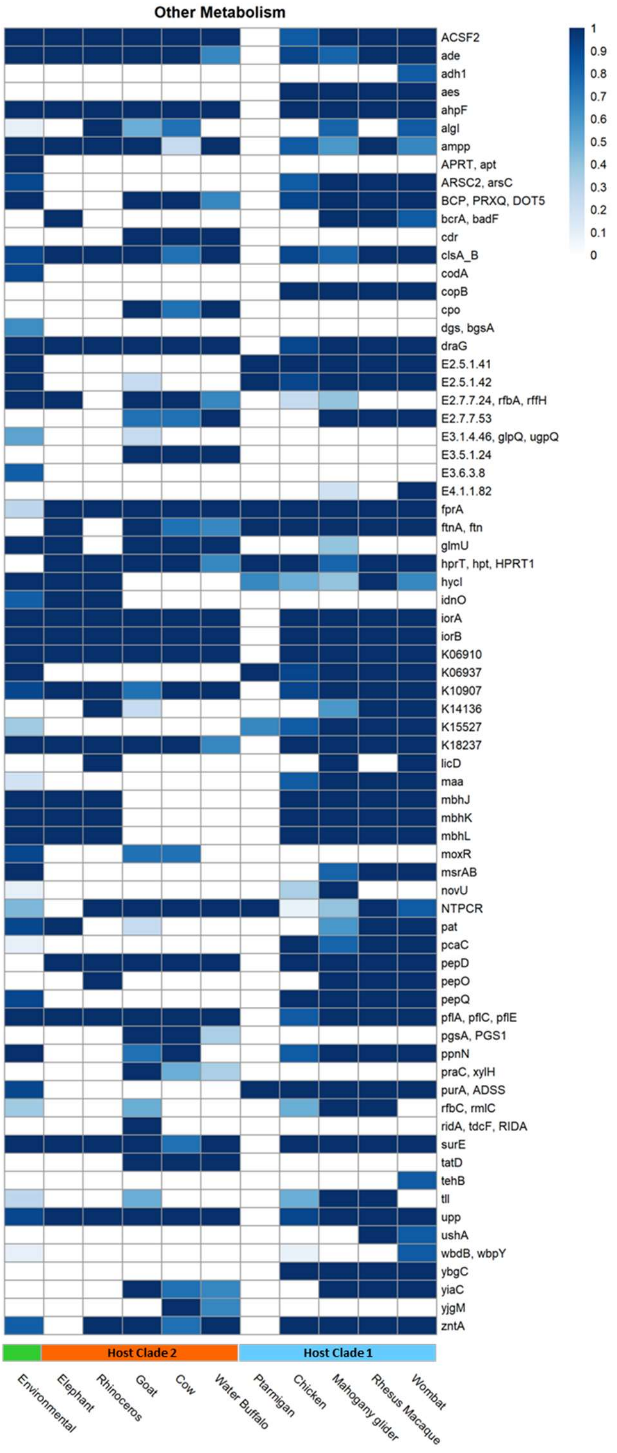

Figure S12. Differential enrichment of other metabolism genes in *Methanocorpusculum*. KO annotation and statistical analyses were performed using the 'annotate' and 'enrichment' functions of EnrichM (v0.4.15; https://github.com/geronimp/enrichM). Genomes were grouped by host species and compared by Fisher's Exact Test, where KOs with corrected p values of <0.05 were retained and considered significant. Heatmap values are colour coded according to the legend and represent the proportion of respective genomes for a given host group. The *Methanocorpusculum* are also labelled as Environmental Clade (green), Host Clade 2 (orange), and Host Clade 1 (blue), as per Figure 3.

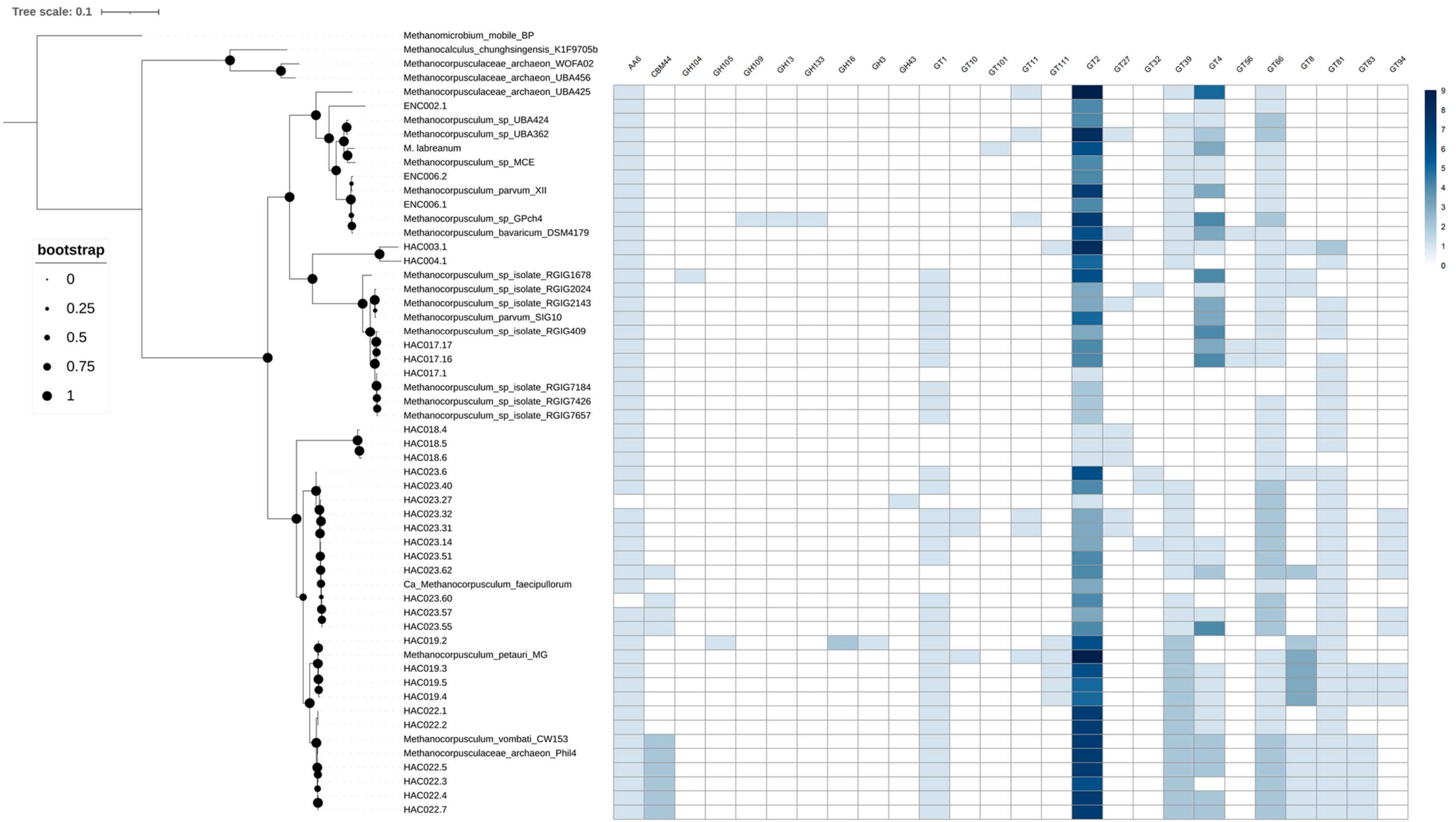

Figure S13. Carbohydrate active enzymes (cazymes) annotated by *Methanocorpusculum* genomes. Cazymes were annotated using DRAM (v1.2.4)(3) and visualised using the pheatmaps (v1.0.12) package in R studio. Values represent individual gene counts of the respective cazymes for

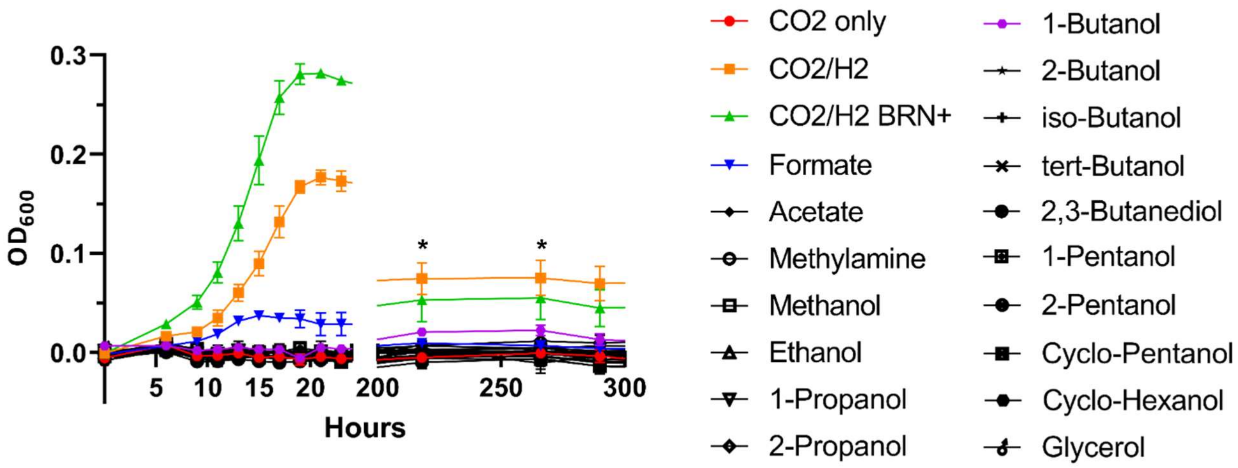

Figure S14. Substrate analysis of *M. petauri* in the presence of CO_2. *M. petauri* was grown with substrates of interest and a headspace of CO_2. No additional substrates showed any significant growth, except for 1-butanol after 200 h which showed a statistically significant increase (max mean OD_{600}= 0.022; P = 0.0032) in yield by OD_{600} compared to CO_2 only. * denotes a p value <0.05.

Figure References

1. Kim D, Park S, Chun J. Introducing EzAAI: a pipeline for high throughput calculations of prokaryotic average amino acid identity. Journal of Microbiology. 2021;59(5):476-80.
2. Chaudhari NM, Gupta VK, Dutta C. BPGA- an ultra-fast pan-genome analysis pipeline. Scientific reports. 2016;6(1):24373.
3. Shaffer M, Borton MA, McGivern BB, Zayed AA, La Rosa Sabina L, Solden LM, et al. DRAM for distilling microbial metabolism to automate the curation of microbiome function. Nucleic acids research. 2020;48(16):8883-900.
4. Kumar S, Stecher G, Li M, Knyaz C, Tamura K. MEGA X: Molecular Evolutionary Genetics Analysis across Computing Platforms. Molecular biology and evolution. 2018;35(6):1547-9.
